# Fungal Community Composition at the Last Remaining Wild Site of Yellow Early Marsh Orchid (*Dactylorhiza incarnata* ssp. *ochroleuca*)

**DOI:** 10.3390/microorganisms11082124

**Published:** 2023-08-21

**Authors:** Andrea Dove, Michael D. Charters, Matthew J. Campbell, Hanna Blake, Manoj Menon, Viswambharan Sarasan

**Affiliations:** 1 Royal Botanic Gardens, Kew, Richmond TW9 3DS, UK; 2 Department of Geography, University of Sheffield, Sheffield S10 2TN, UK; h.blake2001@gmail.com (H.B.);

**Keywords:** orchid mycorrhizal fungi, conservation, native orchid, reintroduction

## Abstract

The yellow early marsh orchid (*Dactylorhiza incarnata* ssp. *ochroleuca*) is a critically endangered terrestrial orchid in Britain. Previous attempts to translocate symbiotic seedlings to a site near the last remaining wild site demonstrated some success, with a 10% survival rate despite adverse weather conditions over a two-year period. However, to facilitate future reintroduction efforts or conservation translocations, a more comprehensive understanding of the fungal microbiome and abiotic soil characteristics at the final remaining wild site is required. Obtaining comprehensive information on both the fungal community and soil nutrient composition from wild sites has significant benefits and may prove critical for the success of future conservation translocations involving threatened orchids. This preliminary study, conducted at the last remaining wild site, revealed a significant correlation between the relative abundance of the orchid mycorrhizal fungal order Cantharellales and the concentrations of nitrate and phosphate in the soil. Another orchid mycorrhizal fungal group, Sebacinales, was found to be distributed extensively throughout the site. The composition of fungal communities across the entire site, orchid-hosting and non-orchid-hosting soils is discussed in relation to reinforcing the current population and preventing the extinction of this orchid.

## 1. Introduction

Despite being the second-largest family of flowering plants, members of the Orchidaceae face a high risk of extinction, with terrestrial orchids being particularly vulnerable [1]. Various factors influence the population dynamics of orchids, including pollinators, climate change, and orchid mycorrhizal fungi [2,3,4,5,6,7,8]. In the wild, successful seedling recruitment of terrestrial orchids depends on the presence of compatible mycorrhizal fungi in the soil, either specialist or generalist [9,10]. Due to their minute, nutrient-lacking seeds, orchid germination relies on the assistance of fungi that provide essential resources such as carbon [11]. Human activities have long-term effects on mycorrhizal fungal communities, contributing to the rarity of terrestrial orchids in critical ecosystems [12,13,14]. Previous studies on mycorrhizal fungi and other fungal groups have shown that declines in orchid populations can be linked to soil characteristics [15,16,17,18,19,20]. While specific relationships have yet to be thoroughly investigated, evidence suggests that variations in mycorrhizal communities driven by habitat conditions impact the local distribution of terrestrial orchids, as seen in genera such as *Dactylorhiza* [21,22,23].

Conservation efforts for orchids require tailored approaches specific to individual species and their ecology [1,24,25,26]. This is particularly relevant for the critically endangered yellow early marsh orchid (*Dactylorhiza incarnata* (L.) *Soó* subsp. *ochroleuca* (*Wüstnei ex Boll*) *P.F.Hunt & Summerh*) [21]. The last remaining population in Britain was found on a protected fen habitat in Suffolk [27], meaning that this orchid is on the brink of extinction. As part of a pilot study, symbiotic seedlings were successfully produced and translocated to a newly identified site near the wild location, aiming to explore the feasibility of conservation translocation for this taxon [27]. Despite facing unexpected challenges such as year-long flooding and an unusually hot and dry summer in 2022, a 10% recovery rate was achieved, with the translocated plants still thriving at the new site [27]. The senility of the remaining population has hindered natural seedling recruitment, and annual fluctuations in orchid numbers at the wild site further emphasise the need for future reinforcement. Consequently, a pragmatic solution is to introduce seedlings to reinforce the last remaining wild site, to regions of the site that currently lack orchids. To ensure success, it is crucial to gain a comprehensive understanding of the mycorrhizal communities at the wild site, including how they vary spatially and in response to biotic conditions.

High-throughput sequencing methods offer significant advantages over conventional approaches in identifying fungal communities within the soil, providing enhanced resolutions and species detection capabilities. Primarily, qPCR and DNA metabarcoding techniques enable the identification and relative quantification of community components, thereby offering valuable insights into fungal community ecology [10,28]. These methods are increasingly becoming primary tools for the assessment of diverse groups of plant-associated fungal communities [29]. As these fungal groups can play a critical role in the fitness of their host plants, understanding their diversity and relative abundance in soils holds great importance for the conservation of threatened orchids.

The aim of this study was to investigate the composition of orchid mycorrhizal fungi (OMF) and key endophytes known to associate with orchids, in regions of the wild site in which orchids were present and not present. To achieve this, soil samples were collected from around orchid populations and across the wider wild site, allowing for a comprehensive analysis of soil biotic and abiotic characteristics. We hypothesised that the OMF diversity and abundance may differ in the proximity of orchid populations, although the absence of such trends could indicate the suitability of the wider site for the reintroduction of orchids. Here, we discuss the compositions of the major fungal group communities and soil characteristics at the last wild site of an endangered orchid in Britain.

## 2. Methods

### 2.1. Sampling Site

**Figure 1:**
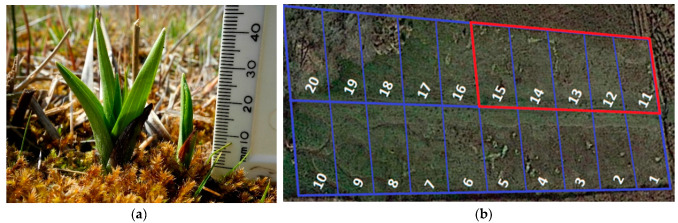
(**a**) Example of yellow early marsh orchid recorded at Britain’s final wild site in April 2021. (**b**) Top-down view of the site divided into 20 × 10 m plots. Orchid-hosting plots are marked in red and non-orchid-hosting plots are marked in blue.

In the spring of 2021, the last remaining wild site of the yellow early marsh orchid (*D. incarnata* ssp. *ochroleuca*), in Suffolk, Britain [27], was visited for data collection. The site was visually surveyed to identify the presence of orchids and subsequently divided into 20 rectangular plots of equal size (Figure 1b). Plots 11–15 were found to have actively growing orchid populations at the time of sampling, while the remaining plots did not.

To collect representative soil samples, five random soil subsamples weighing 20 g each were taken from the upper 5 cm of substrate within each plot. These subsamples were then combined in labelled plastic bags and homogenised. Within 12 h of sampling, soil was transferred to a 4 °C fridge to minimise DNA degradation. For metabarcoding analysis, a small amount of soil (<250 mg) was added to BashingBead™ Lysis Tubes (Zymo Research, Cambridge Bioscience, Cambridge, UK) to preserve the environmental DNA for extraction and amplification processes.

### 2.2. Soil Processing and Chemical Analyses

Fresh soil samples from individual plots were divided into two subsamples and the first subsample was used for water content. The remaining subsamples were air-dried and passed through a 2 mm sieve before the following chemical analyses. Soil pH and electrical conductivity (EC) were measured in triplicate using calibrated HANNA HI8424 pH and EXTECH EC400 EC meters (Camlab, Cambridge, UK).

Soil nitrate was measured using calorimetry (cadmium reduction method). This process involved two stages of adding reagents. First, the NitraVer 6 was added to the diluted soil extract. After the reaction, the NitriVer 3 was added, and the colour intensity of the resultant solution was measured using a calibrated Hach DR900 (Camlab, Cambridge, UK), with a measurement wavelength of 520 nm; calibration involved the use of a blank sample of deionised water.

Colorimetry was used to analyse phosphorus (USEPA Ascorbic Acid Method). This method is a one-stage process, adding PhosVer3 to the soil extract. The intensity of the colour of the resultant solution was measured using a calibrated Hach DR900 with the measurement wavelength of 610 nm. Calibration involved the use of a blank sample of deionised water.

### 2.3. Soil DNA Extraction

DNA was extracted and purification was performed on soil samples stored in BashingBead™ Lysis Tubes using Quick-DNA^TM^ Fecal/Soil Microbe Miniprep Kits (Zymo Research), following the manufacturer’s instructions. Briefly, the soil samples were disrupted using a tissue lyser (QIAGEN, Manchester, UK) for approximately 3 min at 25 Hz. The resulting mixture was then centrifuged, and the supernatant was transferred to Zymo-Spin™ III-F Filter Tubes. Genomic lysis buffer was added to the filtrate and the solution was passed through a Zymo-Spin™ IICR Column. The column was cleaned using DNA Pre-Wash Buffer and g-DNA Wash Buffer and the eluted DNA was passed through a pre-prepped Zymo-Spin™ III-HRC Filter.

The DNA concentration and quality of all 20 eluted samples were assessed using a Nanodrop 2000/2000c Spectrophotometer (Thermo Scientific, Waltham, MA, USA).

### 2.4. Metabarcoding

Purified DNA samples were amplified by PCR in the internal transcribed spacer 2 (ITS2) region, targeting fungi as part of the eDNA survey—fungi pipeline. The analysis included 3 replicate PCRs per sample, with the primers used in the metabarcoding step originating as described by White 1990 [30]. PCRs were performed in the presence of both negative and positive control samples (a mock community with a known composition). Amplification success was determined by gel electrophoresis. PCR replicates were pooled and purified, and sequencing adapters were successfully added and confirmed by gel electrophoresis. Sequences were then quantified using a Qubit broad range kit (Thermofisher, Swindon, UK) according to the manufacturer’s protocol. The final library was sequenced using an Illumina MiSeq V3 kit (San Diego, CA, USA) at 10.5 pM with a 20% PhiX spike in. Resulting sequence data underwent processing using a specialised bioinformatics pipeline, which involved data filtering and trimming, merging paired ends, eliminating sequencing errors (e.g., chimeras), clustering similar sequences into molecular Operational Taxonomic Units (OTUs), and aligning a representative sequence from each cluster with a reference database. These steps transformed raw sequence data into usable data for ecological analysis.

Sequences were demultiplexed based on the combination of the i5 and i7 index tags with bcl2fastq (v2.20.0.422; https://support.illumina.com/sequencing/sequencing_software/bcl2fastq-conversion-software.html (accessed on 15 August 2023)). Paired-end FASTQ reads for each sample were merged with USEARCH v11 [31], requiring a minimum overlap of 80% of the total read length. Merged sequences were quality filtered with USEARCH to retain only those with an expected error rate per base of 0.01 or below and dereplicated by sample, retaining singletons. Dereplicated sequences were then processed with ITSx (v1.1b1) to extract only fungal ITS2 sequences, removing the primers and any remaining ribosomal sequence. Unique ITS2 sequences from all samples were denoised in a single analysis with UNOISE [32], requiring retained zero-radius OTUs (ZOTUs) to have a minimum abundance of eight in at least one sample. Taxonomic assignments were made using sequence similarity [33,34] searches of the ZOTU sequences against two reference databases—the NCBI nucleotide (NCBI nt; downloaded 28 September 2021; https://www.ncbi.nlm.nih.gov/nuccore/ (accessed on 28 September 2021)) database and UNITE (v8.2). Hits were required to ensure a minimum e-score of 1 × 10^−20^ and cover at least 90% of the query sequence.

Consensus taxonomic assignments were made for each OTU using sequence similarity searches against the NCBI nt (GenBank) reference database and UNITE (v8.2). Assignments were made to the lowest possible taxonomic level where there was consistency in the matches. Conflicts were flagged and resolved manually. Minimum similarity thresholds of 98%, 95%, and 92% were used for species-, genus-, and higher-level assignments, respectively. In cases where there were equally good matches to multiple species, public records from GBIF were used to assess which were most likely to be present in the United Kingdom.

In cases where resolution was not possible, higher-level taxonomic identifications or multiple potential identifications were provided. Subsequently, the OTU table underwent filtering to exclude low-abundance OTUs from each sample, using a threshold of <0.025% or <10 reads, whichever was greater. Sequences that were unidentified, non-target, and common contaminants (e.g., human and livestock DNA) were then eliminated. It is important to note that unidentified or misidentified taxa can arise from incomplete or inaccurate reference databases, and some taxa may be missed due to low-quality DNA, environmental contaminants, or the prevalence of other species in the sample.

The identification associated with each hit was converted to match the GBIF taxonomic backbone (3 March 2021 edition; downloaded from https://hosteddatasets.gbif.org/datasets/backbone/2021-03-03/ (accessed on 28 September 2021)), to allow results from different databases to be combined.

Consistency in matches determined the lowest taxonomic level for assignments, with identifications based on fewer than three hits flagged as tentative. Minimum similarity thresholds of 98%, 95%, and 92% were applied for species-, genus-, and higher-level assignments, respectively. To cluster ZOTUs, a 97% similarity threshold was used with the USEARCH tool. An OTU-by-sample table was then generated by mapping dereplicated reads for each sample to the representative sequences of the OTUs, using USEARCH at an identity threshold of 97%. Low-abundance detections were subsequently removed from the analysis. Filter thresholds were established as a percentage of the total reads per sample, utilizing either <0.025% or <10 reads, whichever value was greater. Values in the resulting OTU table were calculated in a comparable manner but expressed as percentages. Each column (sample) represented a sum of 100, with the values indicating the percentage of reads obtained for each OTU sequence in the respective sample.

### 2.5. Statistical Analysis

Pearson correlations (two-tailed with a confidence interval set at 95%) were computed between soil parameters (pH, EC, nitrate, phosphate), diversity indices (Shannon and Simpson), and fungal orders (abundance) using GraphPad Prism (V9 for Mac). The orders considered for the correlations were Cantharellales, Sebacinales, Thelephorales, Capnodiales, Pleosporales, Chaetothyriales, Helotiales, Pezizales, Hypocreales, Sordariales, Agaricales, Tremellales, and Glomerales. The family-level analysis for correlation with soil characteristics was impossible due to insufficient data for all the key fungal groups. Heatmaps with Pearson’s ‘r’ ranging from +1 (blue) to −1 (red) for ‘all plots’, ‘orchid-hosting plots’, and ‘non-orchid-hosting plots’ were prepared.

## 3. Results

### 3.1. Abiotic Soil Characteristics

**Figure 2:**
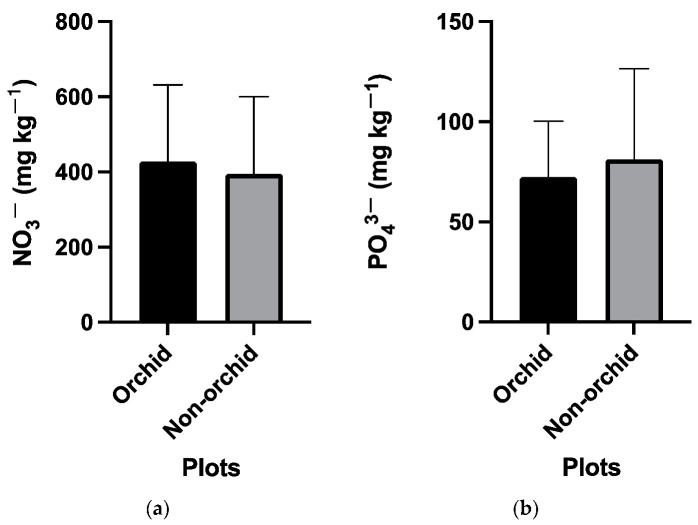
Comparison between orchid-hosting and non-hosting plots on (**a**) nitrate and (**b**) phosphate levels in the 20 plots from the wild site of the yellow early marsh orchid (*D. incarnata* ssp. *ochroleuca*).

While the soil pH was consistent across all plots at the study site, soil phosphate and nitrate concentrations varied spatially (Table 1), with plot 7 exhibiting the highest phosphate and nitrate concentrations (188.92 and 944.59 mg/kg^−1^, respectively) and plot 1 the lowest (15.1 and 75.51 mg/kg^−1^, respectively). However, there were no significant differences in phosphate and nitrate levels between orchid-hosting and non-orchid-hosting plots (Figure 2).

### 3.2. Soil DNA Concentrations and Fungal Community Composition by Plot

**Figure 3:**
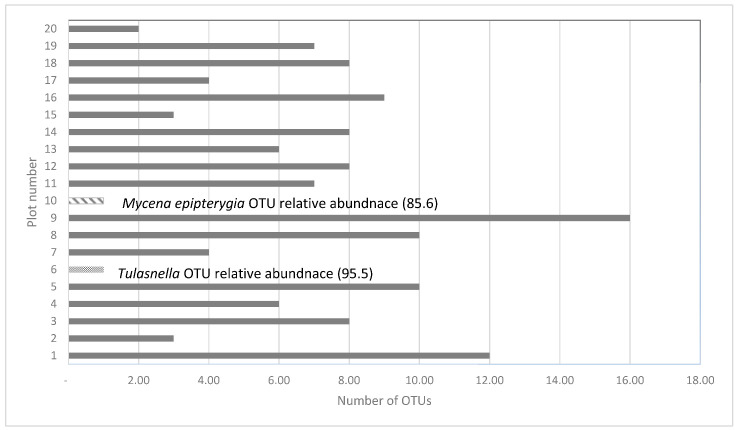
Total number of OTUs recorded in soils from 20 plots at the last wild site of the *D. incarnata* ssp. *ochroleuca*, covering the key families of fungi (Thelephoraceae, Cortinariaceae, Pezizaceae, Helotiaceae, Ceratobasidiaceae, Psathyrellaceae, Sebacinaceae, Tricholomataceae) known to associate with orchids. *Tulasnella* and *Mycena* are represented by a single OTU each with very high relative abundance. Plots 11–15 represent orchid-hosting plots while no orchids were present in plots 1–10 and 16–20.

The total DNA yields of soils collected in the plots ranged between 42.1 ng/µL and 164 ng/µL (Table S1). Soils from plot 6 yielded the second-lowest DNA concentrations (48.8 ng/µL) as well as the lowest number of fungal taxa (10; Table 2) and presented only a single OTU (*Tulasnella*, relative abundance: 95.5; Figure 3). In contrast, soils in plot 10 had the highest DNA yields (164 ng/µL) but, as in plot 6, only a single OTU was identified (*Mycena epipterygia*, relative abundance: 85.6; Figure 3). Although plot 9 yielded the lowest concentration of soil DNA, it exhibited the highest number of OTUs (16; Figure 3), while plots 4 and 7 had the greatest richness of fungal taxa (207 and 236 taxa, respectively; Table 2).

In general, mycorrhizal fungal OTUs belonging to the Sebacinaceae family were dominant, followed by Thelephoraceae. Sebacinaceae OTUs were detected in all plots except 6 and 7 (Table S2). In comparison, Cerabasdiaceae and Tulasnellaceae exhibited a narrower OTU distribution and relative abundance.

At the order level, three out of five orchid-hosting plots contained Cantharellales and 10 out of 15 non-orchid-hosting plots contained Cantharellales, indicating a relatively even distribution between the two groups (Table S2).

**Figure 4:**
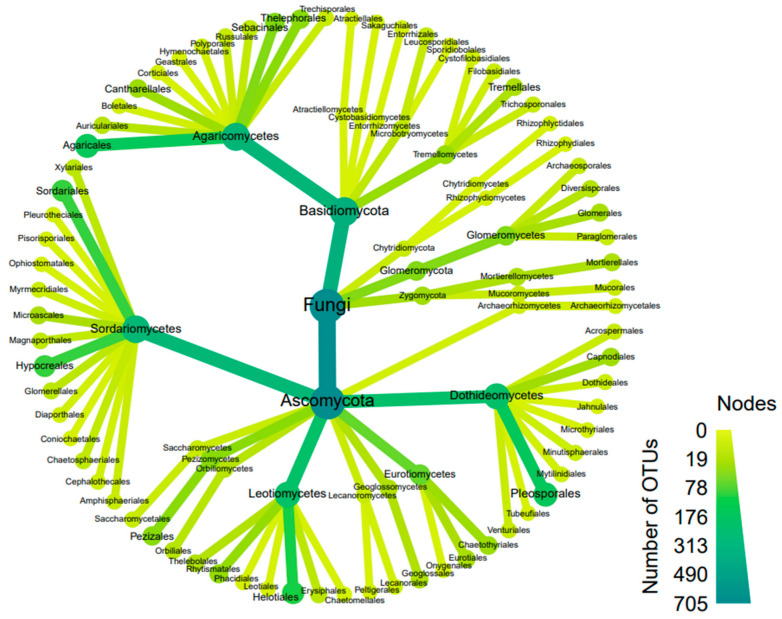
Heat tree showing community composition and relative abundance of different fungal taxonomic groups at order level in the 20 plots from the wild site of the yellow early marsh orchid (*D. incarnata* ssp. *ochroleuca*).

The relative abundance of Agaricales was found to be the highest among all the distributed orders in the site (Table S2). The relative abundance of Agaricales was greater in orchid-hosting plots compared to non-orchid-hosting plots where orchids were absent, with the exception of plot 10. Correlations between larger groups of fungi and soil characterisitcs were conducted at the order level, specifically focusing on the soil nutrients nitrate and phosphate. Pleosporales fungi were the most abundant order and, similar to Capnodiales, were present in all plots. Helotiales and Sordariales exhibited good relative abundance in all plots except for plot 6. Overall, the relative abundance of Ascomycetous fungi (mainly falling into dark septate endophytes) was observed in Pleosporales, Helotiales, Hypocreales, and Sordariales. On the other hand, the most abundant group of Basdiomycetous was Agaricales, followed by Sebacinales and Thelephorales (Figure 4). In plots 5 and 6, the diversity of fungi, including both endo- and ectomycorrhizal fungi, was low. Interestingly, both plots 5 and 6 were colonised by the fungal genus Paraphoma.

Agaricales were detected in all plots except for plot 6. Ectomycorrhizal fungi in the order of Thelephorales were present in most plots except for plots 2, 6, and 20. Among these plots, five exhibited a notably high relative abundance of this fungal group.

### 3.3. Relationship between Soil Characteristics and Fungal Distribution and Relative Abundance

Results are shown for all 20 plots within the site (all plots), orchid-hosting plots (11–15), and non-orchid-hosting plots (1–10 and 16–20). The analysis of the data generated a symmetrical correlation heatmap with Pearson’s ‘r’ ranging from +1 (blue) to −1 (red), and the corresponding *p* values for each correlation are presented in the Supplementary File, Table S3.

#### 3.3.1. All Plots

**Figure 5:**
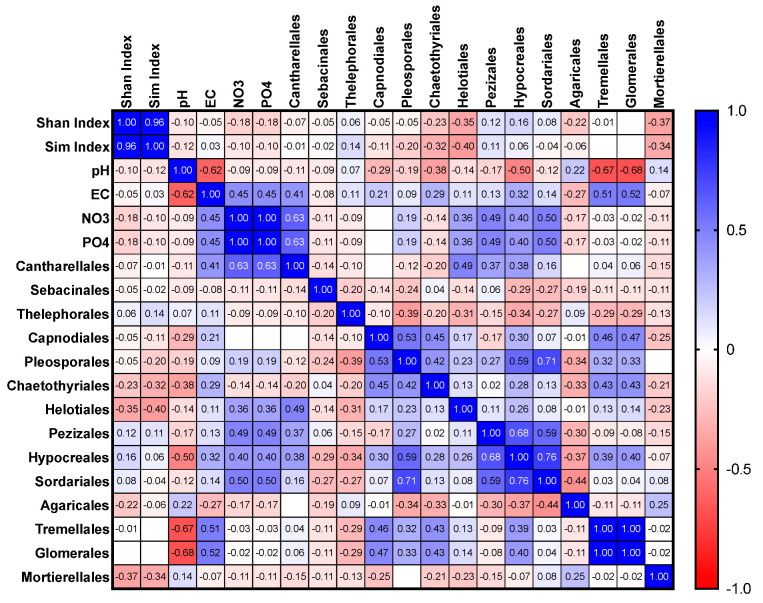
Correlations among selected fungal orders in relation to Shannon and Simpson indices and nitrate and phosphate levels in the 20 plots from the wild site of the yellow early marsh orchid (*D. incarnata* ssp. *ochroleuca*).

**Figure 6:**
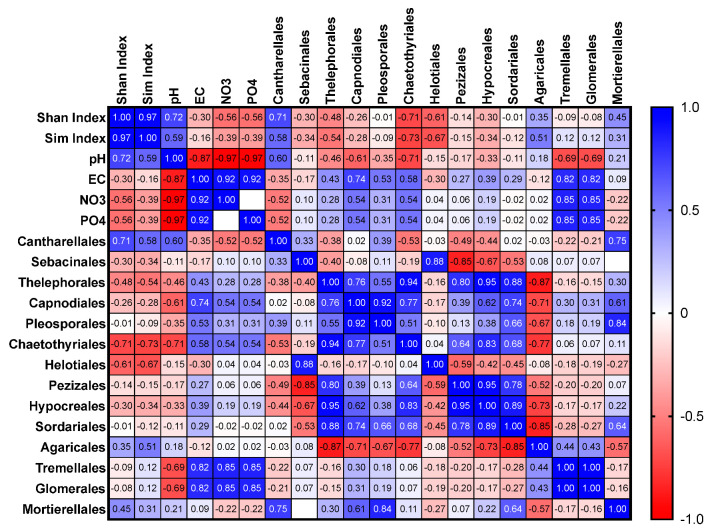
Correlations between different fungal orders in relation Shannon and Simpson indices and nitrate and phosphate levels in orchid-hosting plots from the wild site of the yellow early marsh orchid (*D. incarnata* ssp. *ochroleuca*).

**Figure 7:**
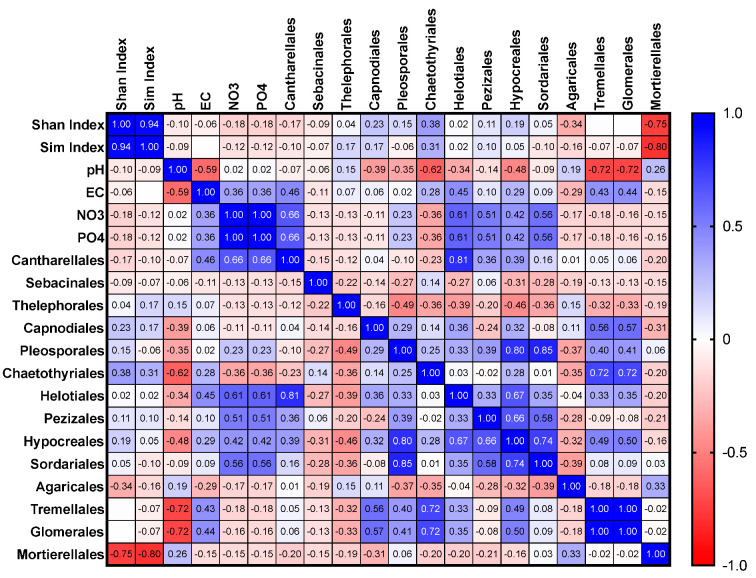
Correlations between different fungal orders in relation to Shannon and Simpson indices and nitrate and phosphate levels in non-orchid-hosting plots from the wild site of the yellow early marsh orchid (*D. incarnata* ssp. *ochroleuca*).

The following orders were thoroughly analysed for correlations with soil nutrients and other conditions: Cantharellales, Sebacinales, Thelephorales, Capnodiales, Pleosporales, Chaetothyriales, Helotiales, Pezizales, Hypocreales, Sordariales, Agaricales, Tremellales, and Glomerales (Figure 5, Figure 6 and Figure 7). The relative abundance of Cantharellales (*p* = 0.003), Pezizales (*p* = 0.029), and Sordariales (*p* = 0.24) showed a positive correlation with nitrate and phosphate across all plots. Furthermore, significant positive correlations were observed for Pezizales and Sordariales with these nutrients (Figure 5). The dominant order Pleosporales exhibited a positive correlation with both Hypocreales (*p* = 0.006) and Sordariales (*p* = 0.0004).

#### 3.3.2. Orchid-Hosting Plots Only

Overall, the distribution of the key OMF orders Cantharellales and Sebacinales was not significantly correlated with soil nutrients such as phosphates and nitrates in the orchid-hosting plots (Figure 6). When comparing the entire wild site, orchid-hosting plots, and non-orchid-hosting plots separately, it was observed that the relative abundance of Hypocreales exhibited a positive correlation with Pezizales within the orchid-hosting plots. Agaricales, the most dominant basidiomycetous fungus, showed a significant negative correlation with Hypocreales (*p* = 0.0448).

#### 3.3.3. Non-Orchid-Hosting Plots

In the non-orchid-hosting plots, the relative abundance of Cantharellales was positively correlated with nitrate and phosphate, which was also observed for Helotiales (Figure 7). Similarly, the relative abundance of Hypocreales displayed positive correlations with Pleosporales, Helotiales, Sordariales, and Pezizales.

## 4. Discussion

The conservation translocation of threatened orchids provides a means to mitigate the risk of extinction within this highly diverse plant family. When working with endangered species, it is common to study small population sizes, as was the case at the final remaining wild site of the yellow early marsh orchid in Britain, which had only 16 wild plants at the time of sampling. In this study, we employed a targeted sampling approach to investigate the abiotic soil conditions and fungal microbiome composition in plots hosting orchids and not hosting orchids. Our goal was to assess whether the site harboured essential groups of orchid mycorrhizal fungi and understand how their diversity and relative abundance varied in relation to orchid populations and soil nutrient concentrations. This information would aid future conservation translocation studies at the same site or new sites.

There were no significant differences in phosphate and nitrate levels between the soils of orchid-hosting plots and non-orchid-hosting plots. This was perhaps to be expected, given that the habitat was a fen with good drainage and dense fen vegetation. While the proportion of total sequence reads does not directly indicate the relative abundance of taxa, it can be assumed that the proportion of sequence reads reflects their abundance. However, total sequence reads can be influenced by factors such as the biomass, soil condition, and type of primer used, among others. Despite some methodological limitations, the relative abundance discussed here is of sufficient quality to compare the sites regarding the varying presence of these fungi in soils sampled from different plots within the wild site.

Only 25% of the entire wild site, divided into 20 plots measuring 20 m by 10 m each, hosted orchids at the time of soil collection. As reported before, it is possible that orchids were present in the past or may appear in the future, as there have been fluctuations in population numbers, although these numbers have been declining over recent decades [21,23]. The wild site is nutrient-rich in comparison to other orchid habitats, where orchids are found in nutrient-poor habitats [14,24,25]. Given that genus- or family-level comparisons only allowed limited comparisons between different plots within the wild site, our analyses were conducted at the order level. When we separately assessed the entire site and non-orchid-hosting plots for the relative abundance of the orchid mycorrhizal fungal order Cantharellales, we found significant positive correlations with nitrate and phosphate levels. However, in orchid-hosting plots, the relative abundance of Cantharellales was negatively correlated with nitrate and phosphate, although not significantly so. These findings contrasted the results from the whole wild site, which were consistent with previous research [13,24].

Overall, the comparisons of plots regarding community composition and relative abundance yielded interesting results. Plots 6 and 10 exhibited the lowest diversity and very high relative abundance of two OMF genera (*Tulasnella* and *Mycena*). These values indicate that the high abundance of these OMFs suppressed the distribution of other fungi, both mycorrhizal and non-mycorrhizal. Despite being non-orchid plots, these plots were adjacent to where orchids were present. The responses of OMFs to ecosystem development remain an emerging area of research [35], and they are not known to dominate ecosystems [36]. It is challenging to infer whether these orchid mycorrhizal fungi have a symbiotic relationship with this orchid based solely on their relative abundance in soil and their suppression of other fungi. Further sampling on a temporal scale is needed to understand the relationship between orchids, fungi, and the soil conditions. The sampling of roots from the last remaining population is restricted due to the small number of plants left in the wild. However, understanding seed-germination-compatible fungi from the wild population will help in understanding the role of the dominant fungi *Tulasnella* and *Mycena* OTUs. The distribution and relative abundance of OMFs were not strictly correlated with the distance from the host orchid, as reported in other studies [7,37]. Furthermore, in our study, mycorrhizal fungi previously identified in orchid roots were either absent or remained undetected in soil [38].

Sebacinales, which are a crucial group of OMFs, have been found to be widely distributed in most landscapes, although they are affected by changes in land use [26,27,28]. Our analysis supports these findings by revealing the presence of Sebacinales in the majority of the wild site plots. Previous studies in natural ecosystems, such as temperate grasslands [29], arctic vegetation [30], and forest soil [31], have reported average read numbers of Sebacinales ranging from 1.7% to 11.3% of all fungi. In our study, this range was between 0.1% and 8.2%. However, the relative abundance was lower in the orchid-hosting plots compared to non-orchid-hosting plots, where it was comparatively better. There was no correlation between Sebacinales and the nutrient levels of nitrates and phosphates.

Previous research has observed a relationship between elevated P content and lower mycorrhizal diversity in a European and Madagascan terrestrial orchid species [13,32]. Mujica et al. [18] suggested that differences in mycorrhizal diversity among the wild sites that they studied were driven by differences in soil P and N content. They also stated that higher soil nutrient availability promotes specialisation in orchid–mycorrhizal associations, particularly in soils with high N availability. The genus *Dactylorhiza*, which is widely distributed in the UK and the rest of Europe, includes taxa such as *D. incarnata* ssp. *ochroleuca*, which are threatened to the point of extinction. As this is a fen habitat, the nutrient content can vary significantly and function as a limiting factor for mycorrhizal fungal diversity and relative abundance across seasons. In a study involving several species of *Dactylorhiza*, Jacquemyn et al. [21] suggested that while orchid mycorrhizal fungi have a broad geographic distribution, their occurrence is influenced by specific habitat conditions.

Considering the challenges posed by climate change and the rapid decline in wild habitats, an evidence-based approach to reintroduction and conservation translocation for threatened orchid species is crucial. Detailed soil studies of wild sites serve as a good starting point, as historical land use changes have led to population declines in many terrestrial orchids. Although our current study focused only on a wild site and soil samples collected during summer, conducting a comprehensive study at different times of the year and across a larger area within the wild site will help us to understand the potential to host more plants in the future. A study found that the success of reintroduced and translocated populations of a terrestrial orchid was influenced by the climate and orchid mycorrhizal abundance [39]. The success of the translocation study performed with this orchid species in 2020 [21] was also influenced by extreme weather conditions over two years. Reinforcement at the wild site, given its better drainage compared to the colonisation site, offers potential benefits to augment the existing population. This is promising news for this critically endangered orchid, as the entire wild site holds potential for future reinforcement efforts.

Our study highlights the intricate relationship between soil abiotic conditions and fungal community composition at Britain’s last wild site of the yellow early marsh orchid. The entire site exhibits a favourable distribution and relative abundance for some of the key fungal groups known to associate with orchids, particularly the genus *Dactylorhiza* [16,38,40,41]. A comparative assessment of wild sites hosting orchids with potential receiver sites in terms of nutrient levels and fungal community composition is critical for reintroduction, the reinforcement of populations, and assisted colonisation. Such research will help to identify nearly optimal sites, as previous studies have demonstrated the impact of seasonality on orchid mycorrhizal fungal community compositions [42,43].

## 5. Conclusions

We found a significant positive correlation between the relative abundance of Cantharellales and nitrate/phosphate levels in the soil. Sebacinales, a widespread orchid mycorrhizal fungal group, dominated the entire site in terms of distribution. Based on our preliminary soil assessment, it can be inferred that the areas within the remaining wild site where orchids are currently absent are suitable for the reinforcement of the existing population.

The utilisation of DNA metabarcoding as a preliminary study presented here provided valuable insights into the community composition and relative abundance of fungi. Further studies conducted over different seasons are essential to facilitate the successful conservation translocation of this orchid. These studies should compare the wild site with potential receiver sites, considering the nutrient levels and fungal community composition. The sampling of roots to identify seed-germination-compatible mycorrhizal fungus/fungi is currently not feasible due to the small number of plants left in the last remaining wild site studied here. Further study, when sufficient samples of roots are available, will help to develop better systems for the recovery of this orchid.

## Figures and Tables

**Table 1:** Soil abiotic characteristics (pH, electric conductivity, soil water content, phosphate, and nitrate) in the 20 plots from the wild site of the yellow early marsh orchid (*D. incarnata* ssp. *ochroleuca*). Coloured rows represent orchid-hosting plots at the time of sampling (shaded red) but were absent from all other plots.

Plot	pH	EC (µS cm^−1^)	PO_4_ (mg kg^−1^)	NO_3_ (mg kg^−1^)	D	H	DNA Yield (ng µL^−1^)
1	7.65	573	15.1	75.51	0.90	2.62	84
2	7.98	312	36.18	180.91	0.50	0.93	70
3	7.87	470	95.97	479.86	0.87	2.27	78
4	7.5	479.33	74.82	374.1	0.38	0.96	90
5	7.81	791	71.47	357.33	0.83	2.09	99.1
6	7.82	830.67	70.48	352.4	0.86	2.18	48.8
7	7.54	1143.67	188.92	944.59	0.65	1.44	87
8	7.64	954.33	102.89	514.47	0.20	0.57	103
9	7.58	634.67	74.7	373.52	0.62	1.38	42.1
10	7.44	753.33	66.25	331.27	0.79	1.58	164
11	7.63	968.33	113.27	566.35	0.50	0.86	57
12	7.85	566.33	53.17	265.87	0.85	2.41	95
13	7.76	418	65.96	329.82	0.04	0.12	140
14	7.88	397.33	43.01	215.03	0.72	1.86	118
15	7.67	829.33	86.08	430.39	0.08	0.24	76
16	7.64	745	151.48	757.42	0.86	2.13	82
17	7.52	655.33	120.1	600.49	0.88	2.41	104
18	7.43	636.67	57.13	285.66	0.88	2.66	104
19	7.06	1271.33	44.59	222.95	0.80	1.89	71
20	7.53	659.33	48.88	244.4	0.92	2.80	91

**Table 2:** Community composition of soil fungi at the last wild site of the yellow early marsh orchid (*D. incarnata* ssp. *ochroleuca*) in Britian, categorised based on taxonomic groups. Coloured rows show orchid-hosting plots while no orchids were present in plots 1–10 and 16–20.

Sample ID	Class	Order	Family	Genus	Taxa (Species)
1	12	33	49	49	173 (20)
2	8	16	24	13	74 (4)
3	11	21	31	25	103 (8)
4	12	38	60	49	207 (26)
5	7	19	22	17	48 (4)
6	4	5	5	4	10 (0)
7	11	36	67	59	236 (25)
8	12	30	42	35	149 (10)
9	10	23	29	23	74 (9)
10	8	17	25	21	82 (10)
11	12	29	39	34	151 (13)
12	10	24	35	36	139 (14)
13	8	19	31	22	99 (5)
14	12	32	39	33	138 (13)
15	12	29	43	35	164 (14)
16	10	25	38	32	146 (12)
17	12	29	43	38	151 (15)
18	12	31	47	44	178 (16)
19	11	25	41	42	171 (22)
20	13	30	44	40	172 (18)

## Supplementary Materials

The following supporting information can be downloaded at: https://www.mdpi.com/article/10.3390/microorganisms11082124/s1, Table S1: DNA yields from 20 soil samples collected from the wild site of *Dactylorhiza incarnata* ssp. *ochroleuca*; Table S2: Metabarcoding results of soil samples of *Dactylorhiza incarnata* ssp. *ochroleuca* collected from 20 sites showing community composition and relative abundance percentages; Table S3: Soil analysis results showing *p* values from Pearson Correlation analysis of all plots studied, orchid hosting plots, and non-orchid hosting plots.
