# Supplementary material for: Fungal Community Composition at the Last Remaining Wild Site of Yellow Early Marsh Orchid (Dactylorhiza incarnata ssp. ochroleuca)
Source: Microorganisms. 2023 Aug 21;11(8):2124. doi: 10.3390/microorganisms11082124 (PMC10459220; doi:10.3390/microorganisms11082124)
Supplement: Supplementary file 1 [file microorganisms-11-02124-s001.zip › microorganisms-2517897-supplementary.pdf]

Table S1

DNA yields from 20 soil samples collected from the wild site of *Dactylorhiza incarnata* ssp. *ochroleuca*

Sample ID	DNA (ng/µl)	260/280	260/230
1	84	1.8	1.5
2	70	1.9	1.4
3	78	1.9	1.1
4	90	1.8	1.4
5	99.1	1.61	0.95
6	48.8	1.87	2.01
7	87	1.7	1.3
8	103	1.9	1.6
9	42.1	1.94	0.83
10	164	1.8	1.4
11	57	1.9	1.5
12	95	1.9	1.5
13	140	1.8	1.1
14	118	1.8	1.3
15	76	1.8	1.5
16	82	1.8	1.5
17	104	1.8	1
18	104	1.8	1.8
19	71	1.8	1.5
20	91	1.7	1.2

Table S2. Metabarcoding results of soil samples of *Dactylorhiza incarnata* ssp. *ochroleuca* collected from 20 sites showing community composition and relative abundance percentages.

Phylum	Class	Order	Family	Genus	Species	1	2	3	4	5	6	7	8	9	10	11	12	13	14	15	16	17	18	19	20
Ascomycota	Dothideomycetes	Capnodiales	Cladosporiaceae			0.88	0.38	3.77	3.65	0.13	1.47	2.08	0.92	0.06	0.35	1.97	2.70	0.32	0.24	1.52	0.74	0.60	1.98	3.54	1.83
Ascomycota	Dothideomycetes	Capnodiales	Cladosporiaceae			-	-	-	-	-	-	-	-	-	-	-	-	0.14	-	-	-	-	-	-	-
Ascomycota	Dothideomycetes	Capnodiales	Mycosphaerellaceae	*Periconiella*		-	-	-	-	-	-	-	-	-	-	-	-	0.04	-	-	-	-	-	-	-
Ascomycota	Dothideomycetes	Capnodiales	Mycosphaerellaceae	*Ramichloridium*		0.16	-	-	0.03	-	-	-	-	-	-	-	-	-	-	-	-	-	-	-	-
Ascomycota	Dothideomycetes	Capnodiales	Mycosphaerellaceae	*Ramularia*		-	-	-	-	-	-	-	-	-	-	-	-	0.04	-	-	-	-	-	-	-
Ascomycota	Dothideomycetes	Capnodiales	Mycosphaerellaceae	*Zymoseptoria*		-	-	-	-	-	-	-	-	-	-	-	-	-	-	-	-	0.05	-	0.09	-
Ascomycota	Dothideomycetes	Capnodiales	Mycosphaerellaceae			0.06	-	0.09	-	-	-	0.07	-	-	-	-	0.13	0.04	-	0.04	-	-	0.23	0.08	0.05
Ascomycota	Dothideomycetes	Capnodiales	Mycosphaerellaceae			-	-	-	-	-	-	0.28	-	-	-	-	-	-	-	-	-	0.09	-	-	-
Ascomycota	Dothideomycetes	Capnodiales	Mycosphaerellaceae			0.26	0.05	0.19	0.15	-	-	0.05	0.19	-	0.04	-	0.24	0.08	-	0.09	0.22	0.26	0.42	0.09	0.05
Ascomycota	Dothideomycetes	Capnodiales	Mycosphaerellaceae			-	-	0.07	0.03	-	-	-	-	-	-	0.07	0.09	-	-	0.08	0.07	-	-	0.07	0.06
Ascomycota	Dothideomycetes	Capnodiales	Mycosphaerellaceae			0.05	-	0.07	-	-	-	-	0.08	-	-	0.07	0.07	-	-	0.09	-	-	0.07	0.16	-
Ascomycota	Dothideomycetes	Capnodiales	Neodevriesiaceae	*Neodevriesia*		-	-	0.24	0.05	-	-	-	-	-	-	0.06	0.09	0.06	-	0.05	0.08	-	0.32	-	-
Ascomycota	Dothideomycetes	Capnodiales	Neodevriesiaceae	*Neodevriesia*		-	-	-	-	-	-	-	-	-	-	-	0.05	-	-	-	-	-	-	-	-
Ascomycota	Dothideomycetes	Capnodiales	Teratosphaeriaceae	*Catenulostroma*	*Catenulostroma hermanusense*	-	-	0.12	-	-	-	-	-	-	1.01	0.22	0.08	0.06	-	-	-	-	0.10	-	-
Ascomycota	Dothideomycetes	Capnodiales	Teratosphaeriaceae	*Devriesia*		0.29	-	0.16	-	-	-	0.04	-	-	0.07	0.84	0.14	0.26	-	0.20	-	-	0.36	0.05	-
Ascomycota	Dothideomycetes	Capnodiales	Teratosphaeriaceae			-	-	-	0.06	-	-	-	-	-	-	-	-	-	-	-	-	-	-	0.05	-
Ascomycota	Dothideomycetes	Capnodiales		*Microcyclospora*	*Microcyclospora tardicrescens*	0.21	-	-	-	-	-	-	-	-	-	-	-	-	-	-	-	-	-	-	-
Ascomycota	Dothideomycetes	Capnodiales				0.93	0.27	0.58	0.10	-	-	-	0.09	-	0.66	0.39	0.39	0.51	-	4.16	0.53	-	0.56	0.48	-
Ascomycota	Dothideomycetes	Capnodiales				0.14	-	-	-	-	-	-	-	-	-	-	-	-	-	0.24	-	-	-	-	-
Ascomycota	Dothideomycetes	Capnodiales				0.09	0.03	0.13	-	-	-	-	-	-	0.04	0.10	0.07	-	-	0.07	-	-	0.23	0.09	-
Ascomycota	Dothideomycetes	Capnodiales				-	-	0.09	-	-	-	-	-	-	-	-	-	-	-	-	-	-	-	-	-
Ascomycota	Dothideomycetes	Capnodiales				-	-	-	-	-	-	0.04	-	-	-	-	-	-	-	-	-	-	-	-	-
Ascomycota	Dothideomycetes	Pleosporales	Amniculicolaceae	*Amniculicola*	*Amniculicola longissima*	0.05	-	-	0.06	-	-	0.60	0.23	-	-	-	-	-	-	-	0.32	0.70	-	-	0.06
Ascomycota	Dothideomycetes	Pleosporales	Cucurbitariaceae			-	-	-	-	-	-	0.25	-	-	-	-	-	-	-	-	-	-	-	-	-
Ascomycota	Dothideomycetes	Pleosporales	Dictyosporiaceae	*Jalapriya*	*Jalapriya toruloides*	-	-	-	-	-	-	0.09	-	-	-	-	-	-	-	-	-	0.05	-	0.05	-
Ascomycota	Dothideomycetes	Pleosporales	Didymellaceae	*Calophoma*	*Calophoma sandfjordenica*	0.13	0.03	-	0.32	-	-	0.28	0.30	-	0.07	0.26	0.74	0.09	0.05	0.22	0.17	0.10	0.16	0.80	0.96
Ascomycota	Dothideomycetes	Pleosporales	Didymellaceae	*Neoascochyta*	*Neoascochyta tardicrescens*	-	-	-	0.64	-	-	0.16	-	-	-	0.11	0.07	-	-	0.06	-	-	-	0.66	-
Ascomycota	Dothideomycetes	Pleosporales	Didymellaceae			-	-	-	0.57	-	-	0.11	-	-	-	0.07	-	-	-	0.04	-	0.04	-	0.06	0.08
Ascomycota	Dothideomycetes	Pleosporales	Didymellaceae			-	0.23	-	-	-	-	-	0.05	-	-	0.10	0.12	-	0.13	-	-	-	0.21	0.32	-
Ascomycota	Dothideomycetes	Pleosporales	Didymellaceae			0.19	0.24	0.07	0.07	-	-	0.15	0.09	-	-	0.19	0.31	-	-	0.09	-	0.04	0.19	0.39	0.19
Ascomycota	Dothideomycetes	Pleosporales	Didymellaceae			-	-	-	-	-	-	-	-	0.09	-	-	-	-	-	-	-	-	-	-	-
Ascomycota	Dothideomycetes	Pleosporales	Didymosphaeriaceae	*Laburnicola*		-	-	-	-	-	-	-	-	0.08	-	-	-	-	-	-	-	-	-	-	-
Ascomycota	Dothideomycetes	Pleosporales	Didymosphaeriaceae	*Laburnicola*		-	-	-	-	-	-	-	-	0.55	-	-	-	-	-	-	-	-	-	-	-
Ascomycota	Dothideomycetes	Pleosporales	Didymosphaeriaceae	*Montagnula*		-	-	-	-	-	-	-	-	-	-	-	-	-	-	-	-	-	-	-	0.09
Ascomycota	Dothideomycetes	Pleosporales	Didymosphaeriaceae			0.07	-	-	-	-	-	-	-	-	-	-	-	-	-	-	-	-	-	-	-
Ascomycota	Dothideomycetes	Pleosporales	Didymosphaeriaceae			0.07	-	1.45	-	-	-	0.14	0.07	-	0.03	0.44	-	-	-	0.11	0.49	-	-	-	0.09
Ascomycota	Dothideomycetes	Pleosporales	Lentitheciaceae	*Darksidea*		-	-	-	-	0.07	-	-	-	-	-	-	-	-	-	-	-	-	-	-	-
Ascomycota	Dothideomycetes	Pleosporales	Lentitheciaceae	*Lentithecium*		-	-	-	-	-	-	-	-	-	0.04	-	-	-	-	-	-	-	-	0.12	-
Ascomycota	Dothideomycetes	Pleosporales	Lentitheciaceae	*Lentithecium*		-	-	-	0.03	-	-	-	-	-	-	-	-	-	-	0.15	-	0.04	-	-	-
Ascomycota	Dothideomycetes	Pleosporales	Lentitheciaceae			-	-	-	-	0.09	-	-	-	-	-	-	-	-	-	-	-	-	-	-	-
Ascomycota	Dothideomycetes	Pleosporales	Leptosphaeriaceae	*Leptosphaeria*		-	-	-	0.09	-	-	-	-	-	-	-	-	-	-	-	-	-	-	-	-
Ascomycota	Dothideomycetes	Pleosporales	Leptosphaeriaceae	*Subplenodomus*	*Subplenodomus valerianae*	-	-	-	-	-	-	-	-	-	-	-	-	-	-	-	-	-	-	-	0.09
Ascomycota	Dothideomycetes	Pleosporales	Leptosphaeriaceae			-	-	0.18	-	-	-	-	-	-	-	-	-	-	-	-	-	-	-	-	-
Ascomycota	Dothideomycetes	Pleosporales	Lindgomycetaceae	*Hongkongmyces*		-	-	-	-	-	-	0.06	-	-	-	-	-	-	-	-	-	-	-	-	-
Ascomycota	Dothideomycetes	Pleosporales	Periconiaceae	*Periconia*		-	-	-	-	-	-	0.13	-	-	-	-	-	-	-	-	-	-	-	-	-
Ascomycota	Dothideomycetes	Pleosporales	Periconiaceae	*Periconia*		-	-	0.07	-	-	-	-	-	-	-	0.06	-	-	-	-	0.07	-	0.10	-	-
Ascomycota	Dothideomycetes	Pleosporales	Periconiaceae	*Periconia*		-	-	-	-	-	-	0.06	-	-	-	-	-	-	-	-	-	-	-	-	-
Ascomycota	Dothideomycetes	Pleosporales	Periconiaceae			-	0.04	-	-	-	-	-	-	-	-	-	-	-	-	-	-	-	-	-	-
Ascomycota	Dothideomycetes	Pleosporales	Periconiaceae			-	-	-	-	-	-	0.05	-	-	-	-	-	-	-	-	-	-	-	-	-
Ascomycota	Dothideomycetes	Pleosporales	Phaeosphaeriaceae	*Neosetophoma*		-	-	0.21	0.13	-	-	0.16	0.67	-	-	-	0.21	-	0.06	0.46	0.15	0.37	0.25	-	0.45
Ascomycota	Dothideomycetes	Pleosporales	Phaeosphaeriaceae	*Neosetophoma*		-	0.04	0.06	0.14	-	-	0.11	0.06	-	-	0.12	0.06	-	-	-	-	0.19	0.07	0.41	0.29
Ascomycota	Dothideomycetes	Pleosporales	Phaeosphaeriaceae	*Ophiosphaerella*	*Ophiosphaerella korrae*	0.13	-	0.16	-	-	-	-	0.15	-	-	-	0.94	-	-	0.80	0.07	2.26	0.19	-	-
Ascomycota	Dothideomycetes	Pleosporales	Phaeosphaeriaceae	*Ophiosphaerella*		3.51	-	-	2.16	-	-	0.79	0.48	-	0.15	-	-	0.13	0.50	1.83	3.19	0.61	0.48	-	2.65
Ascomycota	Dothideomycetes	Pleosporales	Phaeosphaeriaceae	*Ophiosphaerella*		-	-	-	0.09	-	-	-	0.06	-	-	-	-	-	-	-	-	-	-	-	-
Ascomycota	Dothideomycetes	Pleosporales	Phaeosphaeriaceae	*Paraphoma*		0.10	-	-	-	0.19	0.17	-	-	-	0.07	-	-	-	-	-	0.06	-	-	-	0.17
Ascomycota	Dothideomycetes	Pleosporales	Phaeosphaeriaceae	*Phaeosphaeria*	*Phaeosphaeria poagena*	-	-	0.08	-	-	-	-	-	-	0.04	-	0.10	-	-	-	-	-	-	0.07	-
Ascomycota	Dothideomycetes	Pleosporales	Phaeosphaeriaceae	*Septoriella*		-	-	-	-	-	-	0.06	-	-	-	3.06	-	-	-	-	-	-	-	-	-
Ascomycota	Dothideomycetes	Pleosporales	Phaeosphaeriaceae	*Stagonospora*		-	-	-	-	-	-	-	-	-	-	-	1.18	-	-	-	-	-	-	-	-
Ascomycota	Dothideomycetes	Pleosporales	Phaeosphaeriaceae	*Stagonospora*		-	0.06	-	0.49	-	-	0.09	0.08	-	-	0.17	0.10	-	0.08	0.10	0.17	0.64	0.09	0.37	0.22
Ascomycota	Dothideomycetes	Pleosporales	Phaeosphaeriaceae	*Wojnowiciella*	*Wojnowiciella leptocarpi*	-	-	-	-	-	-	-	-	-	-	-	-	0.11	-	-	-	-	-	-	-
Ascomycota	Dothideomycetes	Pleosporales	Phaeosphaeriaceae			0.15	0.23	-	0.05	-	-	0.10	0.17	-	-	5.89	0.05	-	0.07	0.08	0.17	0.39	-	0.15	0.33
Ascomycota	Dothideomycetes	Pleosporales	Phaeosphaeriaceae			0.05	-	-	0.05	-	-	0.09	0.07	-	-	0.04	0.10	-	-	0.09	0.19	1.13	-	0.26	0.10
Ascomycota	Dothideomycetes	Pleosporales	Phaeosphaeriaceae			-	0.37	-	-	-	-	-	-	-	0.23	0.09	0.25	0.07	-	-	0.04	-	0.98	1.01	-
Ascomycota	Dothideomycetes	Pleosporales	Phaeosphaeriaceae			0.04	-	-	-	-	-	0.17	0.12	-	-	-	0.32	0.07	-	0.30	0.80	0.07	0.14	0.20	0.15
Ascomycota	Dothideomycetes	Pleosporales	Phaeosphaeriaceae			-	-	-	-	-	-	-	-	-	-	0.71	-	-	-	-	-	-	-	-	-
Ascomycota	Dothideomycetes	Pleosporales	Phaeosphaeriaceae			-	-	0.26	0.13	-	-	-	0.09	-	-	-	0.46	0.09	-	0.10	0.39	-	0.24	0.14	-
Ascomycota	Dothideomycetes	Pleosporales	Phaeosphaeriaceae			-	-	-	0.27	-	-	-	-	-	-	-	-	-	-	-	-	-	-	-	0.05
Ascomycota	Dothideomycetes	Pleosporales	Phaeosphaeriaceae			0.08	0.04	-	-	-	-	-	-	-	0.04	-	0.45	0.06	-	-	0.05	-	-	0.06	-
Ascomycota	Dothideomycetes	Pleosporales	Phaeosphaeriaceae			-	-	-	0.07	-	-	-	0.08	-	-	-	0.38	-	-	0.07	0.22	-	-	-	-
Ascomycota	Dothideomycetes	Pleosporales	Phaeosphaeriaceae			-	-	-	0.05	-	-	0.07	-	-	-	-	-	-	-	-	-	0.12	-	-	0.14
Ascomycota	Dothideomycetes	Pleosporales	Phaeosphaeriaceae			-	-	-	0.16	-	-	-	-	-	-	-	-	-	-	-	-	-	-	-	-
Ascomycota	Dothideomycetes	Pleosporales	Phaeosphaeriaceae			-	-	-	-	-	-	-	-	-	-	-	-	-	-	-	-	0.04	-	-	0.08
Ascomycota	Dothideomycetes	Pleosporales	Phaeosphaeriaceae			-	-	0.10	0.04	-	-	-	0.08	-	-	-	-	-	-	0.22	-	0.06	0.10	-	-
Ascomycota	Dothideomycetes	Pleosporales	Phaeosphaeriaceae			-	-	-	-	-	-	-	-	-	-	-	-	-	-	-	-	-	-	-	0.06
Ascomycota	Dothideomycetes	Pleosporales	Phaeosphaeriaceae			0.12	-	-	-	-	-	-	-	-	-	-	-	-	-	0.07	-	-	-	-	-
Ascomycota	Dothideomycetes	Pleosporales	Phaeosphaeriaceae			-	-	-	-	-	-	-	-	-	-	0.05	-	-	-	-	-	-	-	-	-
Ascomycota	Dothideomycetes	Pleosporales	Phaeosphaeriaceae			-	-	-	0.06	-	-	-	-	-	-	0.11	-	-	-	-	-	-	-	-	-
Ascomycota	Dothideomycetes	Pleosporales	Phaeosphaeriaceae			-	-	0.10	-	-	-	-	-	-	-	-	-	-	-	-	-	-	-	-	-
Ascomycota	Dothideomycetes	Pleosporales	Phaeosphaeriaceae			0.08	-	-	0.03	-	-	-	-	-	-	-	-	-	-	-	-	-	-	-	-
Ascomycota	Dothideomycetes	Pleosporales	Pleomassariaceae			-	-	-	0.05	-	-	0.09	-	-	-	-	-	-	-	-	-	-	-	-	-
Ascomycota	Dothideomycetes	Pleosporales	Pleosporaceae	*Alternaria*		-	-	0.10	0.07	-	-	-	-	-	-	-	-	-	-	-	-	-	-	0.09	-
Ascomycota	Dothideomycetes	Pleosporales	Pleosporaceae	*Alternaria*		-	-	-	-	-	-	0.04	-	-	-	-	-	-	-	-	-	-	-	0.07	-
Ascomycota	Dothideomycetes	Pleosporales	Pleosporaceae	*Pyrenophora*		-	-	-	0.10	-	-	-	-	-	-	-	-	-	-	-	-	-	-	-	-
Ascomycota	Dothideomycetes	Pleosporales	Pleosporaceae			0.04	0.03	0.33	5.17	-	-	0.16	0.08	-	0.04	1.24	0.30	0.09	-	0.10	0.05	0.18	0.19	0.15	0.18
Ascomycota	Dothideomycetes	Pleosporales	Pleosporaceae			-	-	-	-	-	-	0.04	0.05	-	-	0.31	-	-	-	-	-	-	-	-	-
Ascomycota	Dothideomycetes	Pleosporales	Pleosporaceae			-	-	-	-	-	-	-	-	-	-	-	-	-	-	-	-	-	0.06	-	-
Ascomycota	Dothideomycetes	Pleosporales	Pyrenochaetopsidaceae			-	-	-	-	-	-	-	-	-	-	-	-	-	-	-	-	0.13	-	-	0.11
Ascomycota	Dothideomycetes	Pleosporales	Sporormiaceae	*Preussia*	*Preussia terricola*	-	-	-	-	-	-	-	-	-	-	-	-	-	-	-	-	-	-	0.07	-
Ascomycota	Dothideomycetes	Pleosporales	Sporormiaceae	*Preussia*		0.06	-	-	0.35	-	-	0.05	-	-	-	-	-	-	0.26	-	-	0.17	-	-	-
Ascomycota	Dothideomycetes	Pleosporales	Sporormiaceae	*Preussia*		-	-	-	-	-	-	-	-	-	-	-	-	-	-	-	-	0.81	-	-	-
Ascomycota	Dothideomycetes	Pleosporales	Tetraplosphaeriaceae			0.12	-	-	-	-	-	-	-	-	-	-	-	-	-	-	-	-	-	-	-
Ascomycota	Dothideomycetes	Pleosporales	Torulaceae	*Dendryphion*		-	-	-	0.03	-	-	-	-	-	-	-	-	-	-	-	-	-	-	-	-
Ascomycota	Dothideomycetes	Pleosporales	Torulaceae			-	-	-	-	-	-	0.03	-	-	-	-	-	-	-	-	-	-	-	-	-
Ascomycota	Dothideomycetes	Pleosporales		*Pseudorobillarda*	*Pseudorobillarda phragmitis*	-	-	-	-	-	-	-	-	-	-	-	-	-	-	-	0.05	-	0.07	-	-
Ascomycota	Dothideomycetes	Pleosporales				0.07	-	-	2.24	-	-	-	-	-	-	-	-	-	-	-	-	11.55	-	-	-
Ascomycota	Dothideomycetes	Pleosporales				5.16	0.89	2.54	0.20	-	-	0.61	17.48	-	-	0.68	6.74	5.28	2.16	2.65	2.79	0.49	2.32	1.20	0.46
Ascomycota	Dothideomycetes	Pleosporales				0.08	0.45	2.10	3.57	-	-	1.49	0.60	-	0.13	4.17	8.66	0.10	0.60	14.87	0.06	1.13	0.28	2.28	1.39
Ascomycota	Dothideomycetes	Pleosporales				-	0.22	2.19	0.47	-	-	-	-	-	-	-	0.44	6.29	0.38	-	2.16	0.31	1.40	0.60	-
Ascomycota	Dothideomycetes	Pleosporales				2.46	0.66	1.29	0.03	-	-	-	0.33	-	0.55	0.16	8.51	1.30	-	8.24	7.22	0.14	4.05	5.64	6.01
Ascomycota	Dothideomycetes	Pleosporales				-	-	-	-	-	-	-	-	-	-	-	0.09	-	-	0.17	0.14	4.12	-	-	0.09
Ascomycota	Dothideomycetes	Pleosporales				2.45	0.12	0.69	1.84	0.13	-	2.97	0.98	0.06	0.12	0.34	0.73	0.15	0.50	1.08	1.63	4.07	1.13	0.48	1.75
Ascomycota	Dothideomycetes	Pleosporales				0.11	1.69	0.15	-	-	-	-	-	-	0.09	-	-	0.08	-	-	0.07	-	0.68	-	0.51
Ascomycota	Dothideomycetes	Pleosporales				-	-	-	-	-	-	-	-	-	-	-	-	0.08	0.07	-	-	-	-	-	-
Ascomycota	Dothideomycetes	Pleosporales				0.12	-	0.15	0.06	-	-	-	0.12	-	0.40	1.09	0.23	0.27	0.06	0.13	0.14	-	1.95	0.41	0.70
Ascomycota	Dothideomycetes	Pleosporales				0.19	-	-	-	-	-	0.07	0.11	-	-	-	0.16	-	-	0.05	1.81	0.13	-	0.18	0.29
Ascomycota	Dothideomycetes	Pleosporales				0.62	-	0.10	0.20	-	-	0.41	0.58	-	0.07	-	-	-	0.08	0.22	0.10	0.14	-	-	0.74
Ascomycota	Dothideomycetes	Pleosporales				-	-	-	-	-	-	-	-	-	-	-	-	-	-	-	-	-	-	-	0.39
Ascomycota	Dothideomycetes	Pleosporales				-	-	-	-	-	-	-	0.06	-	0.17	-	0.11	0.07	-	0.04	-	-	1.14	0.06	-
Ascomycota	Dothideomycetes	Pleosporales				-	-	0.48	-	-	-	-	0.09	-	0.30	-	0.14	-	-	-	-	-	-	-	-
Ascomycota	Dothideomycetes	Pleosporales				-	-	-	-	-	-	-	0.05	-	-	-	-	-	-	0.13	0.09	0.56	0.45	-	-
Ascomycota	Dothideomycetes	Pleosporales				0.44	0.04	0.11	-	-	-	-	0.43	-	0.10	-	0.12	-	-	0.13	0.68	-	-	-	0.07
Ascomycota	Dothideomycetes	Pleosporales				-	-	-	-	-	-	-	-	-	-	0.07	-	-	0.17	-	-	-	0.24	-	-
Ascomycota	Dothideomycetes	Pleosporales				0.29	-	-	-	-	-	0.05	-	-	-	-	-	-	-	-	-	-	-	-	-
Ascomycota	Dothideomycetes	Pleosporales				-	-	-	0.20	-	-	-	-	-	-	-	-	-	-	-	-	-	-	-	-
Ascomycota	Dothideomycetes	Pleosporales				-	-	-	-	-	-	-	-	-	-	-	-	-	-	-	-	-	-	0.33	-
Ascomycota	Dothideomycetes	Pleosporales				-	-	-	0.12	-	-	0.06	-	-	-	0.09	-	-	-	0.04	-	-	-	0.08	0.10
Ascomycota	Dothideomycetes	Pleosporales				-	-	-	-	0.11	-	-	-	-	-	-	-	-	-	-	-	-	-	-	-
Ascomycota	Dothideomycetes	Pleosporales				-	-	-	-	-	-	-	-	-	-	-	-	-	-	-	-	-	-	-	0.09
Ascomycota	Dothideomycetes	Pleosporales				-	-	-	0.12	-	-	-	-	-	-	0.19	-	-	-	-	-	-	-	-	-
Ascomycota	Dothideomycetes	Pleosporales				-	-	-	-	-	-	-	-	-	-	0.14	-	-	-	-	-	-	-	-	-
Ascomycota	Dothideomycetes	Pleosporales				-	-	-	-	-	-	-	-	-	-	-	-	-	-	-	-	-	-	-	0.07
Ascomycota	Dothideomycetes	Pleosporales				-	-	-	0.06	-	-	-	-	-	-	-	-	-	-	-	-	-	-	-	-
Ascomycota	Dothideomycetes	Pleosporales				-	-	-	-	-	-	0.03	-	-	-	-	-	-	-	-	-	-	-	-	-
Ascomycota	Eurotiomycetes	Chaetothyriales	Herpotrichiellaceae	*Cladophialophora*		-	-	-	-	0.32	-	-	-	0.34	-	-	-	-	-	-	-	-	-	-	-
Ascomycota	Eurotiomycetes	Chaetothyriales	Herpotrichiellaceae	*Cladophialophora*		-	-	-	-	-	-	-	-	-	-	-	-	-	-	-	-	-	0.10	-	-
Ascomycota	Eurotiomycetes	Chaetothyriales	Herpotrichiellaceae	*Exophiala*		-	0.08	-	-	-	-	-	0.08	-	0.06	0.36	0.18	0.41	-	2.25	-	-	0.41	0.64	1.27
Ascomycota	Eurotiomycetes	Chaetothyriales	Herpotrichiellaceae	*Exophiala*		-	-	-	-	0.14	-	-	-	0.15	-	0.14	-	-	0.18	-	0.04	-	-	-	-
Ascomycota	Eurotiomycetes	Chaetothyriales	Herpotrichiellaceae	*Exophiala*		-	0.05	-	-	-	-	-	-	-	-	0.11	-	0.23	-	-	-	-	-	0.38	-
Ascomycota	Eurotiomycetes	Chaetothyriales	Herpotrichiellaceae	*Exophiala*		-	-	-	0.11	-	-	-	-	-	-	-	-	-	-	-	-	-	-	-	-
Ascomycota	Eurotiomycetes	Chaetothyriales	Herpotrichiellaceae	*Exophiala*		0.06	-	-	0.14	-	-	0.03	-	-	-	0.09	-	-	-	-	0.11	-	-	0.37	0.05
Ascomycota	Eurotiomycetes	Chaetothyriales	Herpotrichiellaceae	*Exophiala*		-	-	-	0.05	-	-	-	-	-	-	-	-	-	-	-	-	0.25	-	-	-
Ascomycota	Eurotiomycetes	Chaetothyriales	Herpotrichiellaceae	*Exophiala*		-	-	-	-	-	-	-	-	0.04	-	-	-	-	-	-	-	-	-	-	-
Ascomycota	Eurotiomycetes	Chaetothyriales	Herpotrichiellaceae	*Minimelanolocus*		-	-	-	-	-	-	-	-	-	-	0.12	-	-	-	-	-	-	-	-	-
Ascomycota	Eurotiomycetes	Chaetothyriales	Herpotrichiellaceae	*Minimelanolocus*		0.28	-	-	-	-	-	-	-	-	-	-	-	-	-	-	0.19	0.16	-	-	-
Ascomycota	Eurotiomycetes	Chaetothyriales				-	-	-	-	-	-	-	-	-	-	-	-	0.05	-	-	0.04	-	-	-	-
Ascomycota	Leotiomycetes	Helotiales	Dermateaceae			-	-	-	-	0.26	-	-	-	-	-	-	-	-	-	-	-	-	-	-	-
Ascomycota	Leotiomycetes	Helotiales	Dermateaceae			-	-	-	-	-	-	-	-	-	-	-	-	-	-	0.04	-	-	-	-	-
Ascomycota	Leotiomycetes	Helotiales	Hamatocanthoscyphaceae	*Hyalodendriella*	*Hyalodendriella betulae*	-	-	-	0.16	-	-	0.37	-	-	-	-	-	-	0.07	-	-	0.21	-	-	0.06
Ascomycota	Leotiomycetes	Helotiales	Helotiaceae			0.05	-	-	-	-	-	-	-	-	-	-	-	-	-	-	1.43	-	0.06	0.08	-
Ascomycota	Leotiomycetes	Helotiales	Helotiaceae			-	-	-	-	0.32	-	-	-	0.03	-	-	-	-	-	-	-	-	-	-	-
Ascomycota	Leotiomycetes	Helotiales	Helotiaceae			-	-	-	-	-	-	-	-	-	-	-	-	-	-	-	-	0.03	-	-	-
Ascomycota	Leotiomycetes	Helotiales	Helotiaceae			-	-	-	-	-	-	-	-	-	-	-	-	-	0.05	-	-	-	-	-	-
Ascomycota	Leotiomycetes	Helotiales	Hyaloscyphaceae	*Hyaloscypha*		-	-	-	-	-	-	-	-	0.09	-	-	-	-	-	-	-	-	-	-	-
Ascomycota	Leotiomycetes	Helotiales	Hyaloscyphaceae			-	-	-	-	-	-	-	-	-	-	-	-	0.24	-	-	-	-	-	-	-
Ascomycota	Leotiomycetes	Helotiales	Lachnaceae	*Lachnum*	*Lachnum controversum*	-	-	-	0.08	-	-	-	-	-	-	-	-	-	-	-	-	-	-	-	-
Ascomycota	Leotiomycetes	Helotiales	Lachnaceae	*Lachnum*		-	-	-	-	-	-	-	-	-	-	-	-	-	-	0.13	-	-	-	-	-
Ascomycota	Leotiomycetes	Helotiales	Mollisiaceae	*Phialocephala*	*Phialocephala compacta*	-	-	-	-	-	-	0.09	-	-	-	-	-	-	-	-	-	-	-	-	-
Ascomycota	Leotiomycetes	Helotiales	Mollisiaceae	*Variocladium*		-	-	-	-	-	-	-	-	-	-	-	-	-	-	-	-	0.10	-	-	-
Ascomycota	Leotiomycetes	Helotiales	Mollisiaceae			-	-	-	-	-	-	-	-	-	-	-	-	0.05	-	-	-	-	-	0.22	-
Ascomycota	Leotiomycetes	Helotiales	Ploettnerulaceae			1.54	0.50	0.66	0.58	-	-	20.29	1.38	-	0.35	0.47	0.74	0.48	0.17	1.04	0.45	0.32	1.19	1.89	1.01
Ascomycota	Leotiomycetes	Helotiales	Sclerotiniaceae	*Ciboria*	*Ciboria amentacea*	-	-	-	0.04	-	-	0.08	-	-	-	-	-	-	-	-	-	0.79	-	-	0.29
Ascomycota	Leotiomycetes	Helotiales	Sclerotiniaceae			0.05	-	-	0.05	-	-	0.10	-	-	-	-	-	-	-	-	-	0.04	0.06	0.58	0.12
Ascomycota	Leotiomycetes	Helotiales	Tympanidaceae			-	0.04	-	0.03	-	-	-	-	-	-	3.79	1.41	24.66	-	-	-	-	-	-	-
Ascomycota	Leotiomycetes	Helotiales	Vibrisseaceae	*Acephala*		0.12	-	-	-	-	-	-	0.06	-	-	-	-	-	-	-	-	-	-	-	-
Ascomycota	Leotiomycetes	Helotiales				0.13	-	0.07	0.17	-	-	0.12	0.36	-	0.05	0.20	0.13	0.07	0.07	0.18	0.34	0.27	0.45	1.72	0.26
Ascomycota	Leotiomycetes	Helotiales				0.17	-	0.14	2.70	-	-	0.20	0.71	-	0.09	0.20	0.36	0.04	0.08	2.00	0.82	3.98	0.33	0.68	0.70
Ascomycota	Leotiomycetes	Helotiales				-	2.93	-	-	-	-	-	-	-	-	0.31	0.67	0.52	-	-	0.34	-	1.33	0.05	-
Ascomycota	Leotiomycetes	Helotiales				1.97	0.59	0.89	0.21	0.16	-	0.43	1.21	0.03	0.33	1.25	3.10	1.87	0.27	3.32	5.54	0.39	3.17	2.01	0.72
Ascomycota	Leotiomycetes	Helotiales				-	-	2.53	-	-	-	-	-	-	-	-	-	-	-	-	-	-	0.22	-	-
Ascomycota	Leotiomycetes	Helotiales				0.28	0.11	0.20	-	-	-	0.05	-	-	0.52	0.84	0.12	0.88	0.09	0.09	0.44	-	0.64	1.24	-
Ascomycota	Leotiomycetes	Helotiales				0.10	-	0.12	0.41	-	-	0.21	0.09	-	0.05	0.21	0.15	0.05	-	0.32	-	0.19	0.14	0.36	0.53
Ascomycota	Leotiomycetes	Helotiales				0.09	0.03	-	-	-	-	-	0.05	-	-	-	-	0.11	-	-	0.09	-	0.11	0.14	-
Ascomycota	Leotiomycetes	Helotiales				-	-	-	-	-	-	-	-	-	0.67	-	-	-	-	-	-	-	0.06	0.06	-
Ascomycota	Leotiomycetes	Helotiales				-	-	-	-	-	-	-	-	-	-	-	-	-	-	-	-	-	1.26	0.09	-
Ascomycota	Leotiomycetes	Helotiales				-	-	-	-	-	-	-	-	-	-	-	-	-	-	-	-	-	-	0.07	-
Ascomycota	Leotiomycetes	Helotiales				-	-	-	-	-	-	-	-	-	-	-	-	-	-	-	-	-	-	-	0.48
Ascomycota	Leotiomycetes	Helotiales				-	-	-	0.07	-	-	-	-	-	-	-	-	-	-	-	-	0.06	-	-	-
Ascomycota	Leotiomycetes	Helotiales				-	-	0.26	-	-	-	-	-	-	-	0.11	-	0.30	-	-	-	-	0.23	-	-
Ascomycota	Leotiomycetes	Helotiales				-	-	-	-	-	-	-	-	-	-	-	-	-	-	-	-	-	0.66	-	-
Ascomycota	Leotiomycetes	Helotiales				-	0.15	-	-	-	-	-	-	-	-	-	-	-	-	-	-	-	-	0.08	-
Ascomycota	Leotiomycetes	Helotiales				-	-	-	-	-	-	-	-	-	-	-	-	-	0.20	-	-	-	-	-	-
Ascomycota	Leotiomycetes	Helotiales				-	-	-	-	-	-	-	-	-	-	-	-	-	0.06	-	-	-	-	-	-
Ascomycota	Leotiomycetes	Helotiales				-	0.16	-	0.20	-	-	0.04	-	-	-	-	0.07	0.05	-	-	-	-	0.10	0.08	-
Ascomycota	Leotiomycetes	Helotiales				-	-	-	0.04	-	-	-	-	-	-	-	0.07	-	-	-	-	-	-	-	0.21
Ascomycota	Leotiomycetes	Helotiales				-	-	-	-	-	-	-	0.11	-	-	-	0.09	-	-	0.40	-	-	0.26	-	0.09
Ascomycota	Leotiomycetes	Helotiales				-	-	-	0.03	-	-	-	0.09	-	-	-	-	-	-	-	-	0.05	-	-	0.15
Ascomycota	Leotiomycetes	Helotiales				-	-	-	-	-	-	0.04	-	-	-	-	-	-	0.05	-	-	-	-	-	-
Ascomycota	Leotiomycetes	Helotiales				-	-	-	-	-	-	-	-	-	-	-	-	-	0.19	-	-	-	-	-	-
Ascomycota	Leotiomycetes	Helotiales				-	-	0.12	-	-	-	-	-	-	-	-	-	-	-	-	-	-	-	0.08	-

Ascomycota	Leotiomycetes	Helotiales				-	-	-	-	-	-	-	-	-	-	-	-	-	-	-	-	-	0.08	-	-
Ascomycota	Leotiomycetes	Helotiales				0.06	-	-	-	-	-	-	-	-	-	-	-	-	-	-	-	-	-	-	-
Ascomycota	Leotiomycetes	Helotiales				-	-	-	-	-	-	-	-	-	-	-	-	-	-	-	-	-	0.17	0.07	-
Ascomycota	Pezizomycetes	Pezizales	Ascobolaceae	*Ascobolus*	*Ascobolus denudatus*	-	-	-	-	-	-	0.04	-	-	-	-	-	-	-	-	-	-	-	-	-
Ascomycota	Pezizomycetes	Pezizales	Ascobolaceae	*Ascobolus*		-	-	-	-	-	-	0.27	-	-	-	-	-	-	-	-	-	-	-	-	-
Ascomycota	Pezizomycetes	Pezizales	Ascobolaceae	*Ascobolus*		-	-	-	-	-	-	0.12	-	-	-	-	-	-	-	-	-	-	-	-	-
Ascomycota	Pezizomycetes	Pezizales	Ascobolaceae	*Ascobolus*		-	-	-	-	-	-	-	-	-	-	0.18	-	-	-	-	-	-	-	-	-
Ascomycota	Pezizomycetes	Pezizales	Ascodesmidaceae			-	-	-	-	-	-	0.09	-	-	-	-	-	-	-	-	-	-	-	-	-
Ascomycota	Pezizomycetes	Pezizales	Helvellaceae	*Helvella*	*Helvella crispa*	-	-	-	-	-	-	-	-	0.03	-	-	-	-	-	-	-	-	-	-	-
Ascomycota	Pezizomycetes	Pezizales	Pezizaceae	*Pachyella*		-	-	-	-	-	-	0.06	-	-	-	-	-	-	-	-	-	0.05	-	-	0.10
Ascomycota	Pezizomycetes	Pezizales	Pyronemataceae	*Cheilymenia*		0.06	-	-	-	-	-	-	-	-	-	-	-	-	0.32	-	-	-	-	-	-
Ascomycota	Pezizomycetes	Pezizales	Pyronemataceae	*Humaria*		-	-	-	0.11	-	-	-	-	-	-	-	-	-	-	-	-	-	-	-	-
Ascomycota	Pezizomycetes	Pezizales	Pyronemataceae	*Scutellinia*	*Scutellinia kerguelensis*	-	-	-	-	-	-	0.71	-	-	-	-	-	-	-	-	-	-	-	-	0.05
Ascomycota	Pezizomycetes	Pezizales	Pyronemataceae	*Scutellinia*	*Scutellinia scutellata*	0.17	-	-	-	-	-	0.29	0.13	-	-	-	0.09	-	0.12	0.65	0.22	2.91	-	0.15	0.26
Ascomycota	Pezizomycetes	Pezizales	Pyronemataceae	*Scutellinia*		-	0.04	0.27	-	-	-	-	0.10	-	-	-	-	-	-	-	-	0.13	-	-	-
Ascomycota	Pezizomycetes	Pezizales				-	-	-	-	-	-	-	-	0.95	-	-	-	-	-	-	-	-	-	-	-
Ascomycota	Sordariomycetes	Hypocreales	Bionectriaceae	*Acremonium*	*Acremonium charticola*	-	-	-	-	-	-	-	-	-	-	-	-	-	-	-	-	-	-	0.06	-
Ascomycota	Sordariomycetes	Hypocreales	Bionectriaceae	*Acremonium*	*Acremonium rutilum*	-	-	-	0.04	-	-	-	-	-	-	-	-	-	-	-	-	-	-	-	-
Ascomycota	Sordariomycetes	Hypocreales	Bionectriaceae	*Acremonium*		-	-	-	-	-	-	0.23	-	-	-	-	-	-	-	-	-	-	-	-	-
Ascomycota	Sordariomycetes	Hypocreales	Bionectriaceae	*Hydropisphaera*	*Hydropisphaera erubescens*	-	-	-	-	-	-	-	-	-	-	-	-	-	-	-	-	-	0.32	-	-
Ascomycota	Sordariomycetes	Hypocreales	Bionectriaceae			-	-	-	-	-	-	0.38	-	-	-	-	-	-	-	-	-	-	-	-	-
Ascomycota	Sordariomycetes	Hypocreales	Clavicipitaceae	*Metarhizium*		-	-	-	-	-	-	0.25	-	-	-	-	-	0.08	0.06	-	-	-	0.54	0.06	0.26
Ascomycota	Sordariomycetes	Hypocreales	Clavicipitaceae			-	-	-	-	-	-	-	-	-	-	-	-	-	-	0.04	-	-	-	-	-
Ascomycota	Sordariomycetes	Hypocreales	Cordycipitaceae	*Lecanicillium*		-	-	-	-	-	-	-	-	-	-	-	-	-	-	-	0.08	-	-	-	-
Ascomycota	Sordariomycetes	Hypocreales	Cordycipitaceae			-	-	-	-	-	-	-	-	0.06	-	-	-	-	-	-	-	-	-	-	-
Ascomycota	Sordariomycetes	Hypocreales	Cordycipitaceae			-	-	-	-	-	-	-	-	-	-	-	0.07	-	-	-	-	-	-	-	-
Ascomycota	Sordariomycetes	Hypocreales	Cordycipitaceae			-	-	-	-	-	-	-	-	-	-	-	-	-	-	-	0.05	-	-	-	-
Ascomycota	Sordariomycetes	Hypocreales	Hypocreaceae	*Trichoderma*		-	-	-	-	-	-	-	-	-	-	-	-	-	0.10	-	-	-	-	-	-
Ascomycota	Sordariomycetes	Hypocreales	Hypocreaceae	*Trichoderma*		0.16	-	-	-	-	-	-	-	-	-	-	-	-	-	-	-	-	-	-	-
Ascomycota	Sordariomycetes	Hypocreales	Hypocreaceae			-	-	-	-	-	-	-	-	-	-	-	-	-	0.10	-	-	-	-	-	-
Ascomycota	Sordariomycetes	Hypocreales	Hypocreaceae			-	-	-	-	-	-	-	-	-	-	-	-	-	-	-	-	0.05	-	-	-
Ascomycota	Sordariomycetes	Hypocreales	Hypocreaceae			-	-	-	-	-	-	-	-	-	-	-	-	-	-	-	0.12	-	-	-	-
Ascomycota	Sordariomycetes	Hypocreales	Hypocreaceae			-	-	-	-	-	-	-	-	-	-	-	-	-	0.05	-	-	-	-	-	-
Ascomycota	Sordariomycetes	Hypocreales	Hypocreaceae			-	-	-	-	-	-	0.03	-	-	-	-	-	-	-	-	-	-	-	-	-
Ascomycota	Sordariomycetes	Hypocreales	Nectriaceae	*Dendroclathra*	*Dendroclathra lignicola*	-	-	-	-	-	-	-	-	-	-	0.20	-	-	-	-	-	-	-	2.09	-
Ascomycota	Sordariomycetes	Hypocreales	Nectriaceae	*Mariannaea*		-	-	-	-	-	-	-	-	-	-	-	-	-	-	-	0.34	-	-	-	-
Ascomycota	Sordariomycetes	Hypocreales	Nectriaceae	*Neonectria*		0.06	-	-	-	-	-	0.03	-	-	-	-	-	-	-	-	0.09	0.45	-	-	-
Ascomycota	Sordariomycetes	Hypocreales	Nectriaceae	*Paracremonium*	*Paracremonium variiforme*	-	-	-	-	-	-	0.04	-	-	-	-	-	-	-	-	-	-	-	-	-
Ascomycota	Sordariomycetes	Hypocreales	Nectriaceae	*Stylonectria*		0.07	-	-	-	-	-	-	-	-	-	-	-	-	-	-	-	-	-	-	-
Ascomycota	Sordariomycetes	Hypocreales	Nectriaceae			-	-	-	-	-	-	-	0.05	-	-	-	-	-	-	0.10	-	-	-	-	-
Ascomycota	Sordariomycetes	Hypocreales	Nectriaceae			0.37	0.30	1.60	0.32	-	-	0.20	0.29	-	0.11	0.34	0.38	0.28	0.42	1.72	0.70	2.19	0.96	0.78	0.28
Ascomycota	Sordariomycetes	Hypocreales	Nectriaceae			1.22	0.13	0.29	1.46	-	-	1.55	2.08	-	0.15	0.38	0.30	0.16	0.54	0.73	0.44	1.07	0.61	0.38	2.32
Ascomycota	Sordariomycetes	Hypocreales	Nectriaceae			0.09	-	0.11	0.22	0.08	-	0.13	0.08	0.03	-	-	-	-	0.10	-	0.05	1.13	-	-	-
Ascomycota	Sordariomycetes	Hypocreales	Nectriaceae			-	-	-	0.11	-	-	0.09	-	-	-	-	-	-	0.04	-	-	0.07	-	-	0.10
Ascomycota	Sordariomycetes	Hypocreales	Nectriaceae			-	-	-	0.15	-	-	0.59	-	-	-	-	-	-	0.04	-	-	0.17	-	-	-
Ascomycota	Sordariomycetes	Hypocreales	Nectriaceae			-	-	-	0.07	-	-	-	-	-	-	-	-	-	-	-	-	-	-	-	-
Ascomycota	Sordariomycetes	Hypocreales	Nectriaceae			-	-	-	-	-	-	0.15	-	-	-	0.05	-	-	-	-	-	-	0.08	-	0.06
Ascomycota	Sordariomycetes	Hypocreales	Nectriaceae			-	-	-	-	-	-	0.06	-	-	-	-	-	-	-	-	-	-	-	-	-
Ascomycota	Sordariomycetes	Hypocreales	Nectriaceae			-	-	-	-	-	-	-	-	-	-	-	-	-	-	-	-	0.34	-	-	-
Ascomycota	Sordariomycetes	Hypocreales	Nectriaceae			-	-	-	-	-	-	0.17	-	-	-	-	-	-	0.04	-	-	-	-	-	-
Ascomycota	Sordariomycetes	Hypocreales	Nectriaceae			-	-	-	-	-	-	0.12	-	-	-	-	0.07	-	-	-	-	-	-	-	-
Ascomycota	Sordariomycetes	Hypocreales	Nectriaceae			-	-	-	-	-	-	0.08	-	-	-	-	-	-	-	0.09	-	0.05	-	-	-
Ascomycota	Sordariomycetes	Hypocreales	Nectriaceae			-	-	-	-	-	-	-	-	-	-	-	-	-	-	-	-	-	-	-	-
Ascomycota	Sordariomycetes	Hypocreales	Nectriaceae			-	-	-	-	-	-	0.05	-	-	-	-	-	-	-	-	-	-	-	-	-
Ascomycota	Sordariomycetes	Hypocreales	Nectriaceae			-	-	-	-	-	-	0.04	-	-	-	-	-	-	-	-	-	-	-	-	-
Ascomycota	Sordariomycetes	Hypocreales	Ophiocordycipitaceae			-	-	-	0.03	-	-	-	-	-	-	-	-	-	-	0.04	-	-	0.09	-	-
Ascomycota	Sordariomycetes	Hypocreales	Stachybotryaceae	*Albifimbria*	*Albifimbria verrucaria*	-	-	-	0.13	-	-	0.05	-	-	-	-	-	-	-	-	-	0.05	-	-	0.23
Ascomycota	Sordariomycetes	Hypocreales	Stachybotryaceae			-	-	-	-	-	-	-	-	-	-	-	-	-	-	0.35	-	-	-	-	-
Ascomycota	Sordariomycetes	Hypocreales	Stachybotryaceae			-	-	-	-	-	-	0.06	-	-	-	-	-	-	-	-	-	-	-	-	0.30
Ascomycota	Sordariomycetes	Hypocreales	Stachybotryaceae			-	0.08	-	-	-	-	-	-	-	-	-	-	-	-	-	-	-	-	-	-
Ascomycota	Sordariomycetes	Hypocreales	Stachybotryaceae			-	-	-	-	-	-	0.12	-	-	-	0.05	-	-	-	-	-	-	-	-	-
Ascomycota	Sordariomycetes	Hypocreales	Stachybotryaceae			-	-	-	-	-	-	-	0.21	-	-	-	-	-	-	-	0.04	-	-	-	-
Ascomycota	Sordariomycetes	Hypocreales		*Tricellula*		-	-	-	-	-	-	-	-	-	-	-	-	-	-	0.10	-	-	-	-	-
Ascomycota	Sordariomycetes	Hypocreales				-	-	-	-	-	-	-	-	-	0.14	-	-	-	-	-	-	-	-	-	-
Ascomycota	Sordariomycetes	Hypocreales				0.15	-	-	-	-	-	-	-	-	-	-	-	-	-	0.13	0.16	-	-	-	-
Ascomycota	Sordariomycetes	Hypocreales				0.06	-	-	0.05	-	-	-	0.06	-	-	-	-	-	0.06	-	-	-	0.22	-	-
Ascomycota	Sordariomycetes	Hypocreales				-	-	-	0.04	-	-	0.23	-	-	-	-	-	-	-	-	-	-	-	-	-
Ascomycota	Sordariomycetes	Hypocreales				-	-	-	-	-	-	-	-	-	-	-	-	-	-	-	-	0.25	-	-	-
Ascomycota	Sordariomycetes	Hypocreales				-	-	-	0.09	-	-	-	-	-	-	-	-	-	-	-	-	-	-	-	-
Ascomycota	Sordariomycetes	Hypocreales				-	-	-	0.04	-	-	0.03	-	-	-	-	-	-	-	-	-	-	-	-	-
Ascomycota	Sordariomycetes	Sordariales	Chaetomiaceae	*Chaetomium*		-	-	-	-	0.16	-	-	-	0.06	-	-	-	-	-	-	-	-	-	-	-
Ascomycota	Sordariomycetes	Sordariales	Chaetomiaceae	*Chaetomium*		-	-	-	-	-	-	0.36	-	-	-	-	-	-	-	-	-	-	-	-	-
Ascomycota	Sordariomycetes	Sordariales	Chaetomiaceae	*Chaetomium*		-	-	-	-	-	-	0.22	-	-	-	-	-	-	-	-	-	-	-	-	-
Ascomycota	Sordariomycetes	Sordariales	Chaetomiaceae	*Humicola*		-	-	-	-	-	-	0.05	-	-	-	-	-	-	-	-	-	-	-	-	-
Ascomycota	Sordariomycetes	Sordariales	Chaetomiaceae			-	-	-	-	-	-	0.13	-	-	-	-	-	-	-	-	-	-	-	-	-
Ascomycota	Sordariomycetes	Sordariales	Chaetomiaceae			-	-	-	-	-	-	0.06	-	-	-	-	-	-	-	-	-	-	-	-	-
Ascomycota	Sordariomycetes	Sordariales	Lasiosphaeriaceae	*Apodus*	*Apodus deciduus*	0.53	0.05	-	1.70	-	-	0.64	0.18	-	0.07	0.58	-	-	0.38	0.21	0.10	3.60	0.46	-	0.54
Ascomycota	Sordariomycetes	Sordariales	Lasiosphaeriaceae	*Apodus*		-	-	-	-	-	-	-	-	-	-	-	-	-	-	0.04	-	-	-	-	0.06
Ascomycota	Sordariomycetes	Sordariales	Lasiosphaeriaceae	*Cercophora*	*Cercophora squamulosa*	-	-	-	-	-	-	0.20	-	-	-	-	-	-	-	-	-	-	-	-	-
Ascomycota	Sordariomycetes	Sordariales	Lasiosphaeriaceae	*Cladorrhinum*	*Cladorrhinum flexuosum*	-	-	-	0.18	-	-	-	-	-	-	-	-	-	-	0.24	-	0.06	-	-	-
Ascomycota	Sordariomycetes	Sordariales	Lasiosphaeriaceae	*Podospora*	*Podospora intestinacea*	0.50	-	-	-	-	-	-	-	-	0.04	0.14	0.10	0.06	-	-	0.35	-	0.17	-	0.08
Ascomycota	Sordariomycetes	Sordariales	Lasiosphaeriaceae	*Podospora*		-	-	-	0.98	-	-	0.09	0.76	-	-	-	-	-	0.18	-	-	3.27	-	-	2.99
Ascomycota	Sordariomycetes	Sordariales	Lasiosphaeriaceae	*Podospora*		-	-	-	-	-	-	0.91	-	-	-	-	-	-	-	-	-	-	-	-	-
Ascomycota	Sordariomycetes	Sordariales	Lasiosphaeriaceae	*Podospora*		-	-	1.09	-	-	-	-	-	-	-	-	-	-	0.06	-	-	0.15	-	-	-
Ascomycota	Sordariomycetes	Sordariales	Lasiosphaeriaceae	*Podospora*		0.65	-	-	-	-	-	0.34	0.22	-	-	-	-	-	-	-	-	-	-	-	0.34
Ascomycota	Sordariomycetes	Sordariales	Lasiosphaeriaceae	*Podospora*		0.06	-	-	-	-	-	-	-	-	-	-	-	-	0.08	-	-	-	-	-	0.12
Ascomycota	Sordariomycetes	Sordariales	Lasiosphaeriaceae	*Podospora*		-	-	-	-	-	-	0.06	-	-	-	-	-	-	-	-	0.09	0.06	-	-	-
Ascomycota	Sordariomycetes	Sordariales	Lasiosphaeriaceae	*Schizothecium*	*Schizothecium carpinicola*	0.08	-	0.09	0.04	-	-	-	-	-	-	-	0.27	-	0.20	0.05	-	0.26	-	-	-
Ascomycota	Sordariomycetes	Sordariales	Lasiosphaeriaceae	*Schizothecium*	*Schizothecium carpinicola*	-	-	-	-	-	-	-	-	-	-	-	0.10	-	-	-	-	-	-	-	-
Ascomycota	Sordariomycetes	Sordariales	Lasiosphaeriaceae	*Schizothecium*	*Schizothecium carpinicola*	-	-	-	-	-	-	-	-	-	-	-	-	-	-	-	-	-	-	0.10	-
Ascomycota	Sordariomycetes	Sordariales	Lasiosphaeriaceae	*Schizothecium*		-	-	-	-	-	-	0.47	-	-	-	-	-	-	-	-	-	-	-	-	-
Ascomycota	Sordariomycetes	Sordariales	Lasiosphaeriaceae	*Schizothecium*		0.07	-	-	-	-	-	-	-	-	-	-	-	-	0.05	-	-	-	-	-	-
Ascomycota	Sordariomycetes	Sordariales	Lasiosphaeriaceae	*Zopfiella*		2.18	-	3.22	0.58	-	-	0.32	11.86	-	0.15	-	2.89	-	1.89	0.25	0.31	0.26	0.81	0.14	2.14
Ascomycota	Sordariomycetes	Sordariales	Lasiosphaeriaceae			-	-	-	0.07	-	-	0.09	0.11	-	-	-	-	-	0.07	-	-	0.14	-	-	0.14
Ascomycota	Sordariomycetes	Sordariales	Lasiosphaeriaceae			0.18	0.08	-	-	-	-	0.38	-	-	0.16	-	-	-	-	0.68	1.79	7.39	0.09	0.09	0.60
Ascomycota	Sordariomycetes	Sordariales	Lasiosphaeriaceae			0.20	-	0.10	0.82	-	-	0.08	0.20	-	0.04	0.09	0.48	0.12	0.48	-	5.77	1.68	0.29	0.62	2.73
Ascomycota	Sordariomycetes	Sordariales	Lasiosphaeriaceae			-	-	-	-	-	-	-	-	-	-	-	-	-	-	-	4.54	-	-	-	-
Ascomycota	Sordariomycetes	Sordariales	Lasiosphaeriaceae			0.37	-	-	0.36	-	-	0.21	0.08	-	-	-	0.07	-	-	6.12	-	-	-	-	0.05
Ascomycota	Sordariomycetes	Sordariales	Lasiosphaeriaceae			-	0.04	-	-	-	-	-	0.05	-	-	0.13	1.24	0.09	-	0.48	-	-	-	0.05	-
Ascomycota	Sordariomycetes	Sordariales	Lasiosphaeriaceae			0.48	0.11	-	1.37	-	-	1.11	1.29	-	-	-	0.67	-	0.21	0.48	0.12	1.65	-	0.05	0.85
Ascomycota	Sordariomycetes	Sordariales	Lasiosphaeriaceae			0.06	-	-	-	-	-	1.78	-	-	-	-	-	-	0.05	-	-	0.12	-	-	0.15
Ascomycota	Sordariomycetes	Sordariales	Lasiosphaeriaceae			0.07	-	-	0.09	-	-	0.43	-	-	-	-	-	-	-	2.05	-	0.04	-	-	0.06
Ascomycota	Sordariomycetes	Sordariales	Lasiosphaeriaceae			0.10	-	-	0.13	-	-	0.14	0.56	-	-	0.05	-	-	-	0.15	-	-	0.06	-	-
Ascomycota	Sordariomycetes	Sordariales	Lasiosphaeriaceae			-	-	-	0.68	-	-	-	0.16	-	-	-	-	-	-	-	-	-	-	-	-
Ascomycota	Sordariomycetes	Sordariales	Lasiosphaeriaceae			0.24	-	-	-	-	-	0.28	-	-	-	-	-	-	-	0.05	-	0.72	-	-	0.06
Ascomycota	Sordariomycetes	Sordariales	Lasiosphaeriaceae			-	-	-	0.24	-	-	0.44	0.23	-	-	-	-	-	-	1.31	-	0.06	-	-	0.21
Ascomycota	Sordariomycetes	Sordariales	Lasiosphaeriaceae			0.22	-	-	-	-	-	0.45	-	-	-	-	-	-	-	-	-	0.21	-	-	-
Ascomycota	Sordariomycetes	Sordariales	Lasiosphaeriaceae			-	-	-	-	-	-	-	-	-	-	0.57	-	0.05	-	-	-	-	-	-	-
Ascomycota	Sordariomycetes	Sordariales	Lasiosphaeriaceae			-	0.06	0.49	-	-	-	0.12	-	-	-	-	-	-	-	-	0.04	-	-	-	-
Ascomycota	Sordariomycetes	Sordariales	Lasiosphaeriaceae			-	-	-	-	-	-	0.16	-	-	-	-	-	-	-	-	-	-	-	-	-
Ascomycota	Sordariomycetes	Sordariales	Lasiosphaeriaceae			-	-	-	-	-	-	-	-	-	-	-	-	-	-	-	-	-	-	-	0.10
Ascomycota	Sordariomycetes	Sordariales	Lasiosphaeriaceae			-	-	-	-	-	-	0.12	-	-	-	-	-	-	-	-	-	-	-	-	-
Ascomycota	Sordariomycetes	Sordariales	Lasiosphaeriaceae			-	-	-	-	-	-	-	-	-	-	-	-	-	0.07	-	-	-	-	-	-
Ascomycota	Sordariomycetes	Sordariales	Lasiosphaeriaceae			-	-	-	0.04	-	-	0.06	-	-	-	-	-	-	-	-	-	-	-	-	0.08
Ascomycota	Sordariomycetes	Sordariales	Lasiosphaeriaceae			0.07	-	-	-	-	-	-	-	-	-	-	-	-	-	0.05	0.12	-	-	-	-
Ascomycota	Sordariomycetes	Sordariales	Sordariaceae			-	-	-	-	-	-	0.60	-	-	-	-	-	0.22	-	-	-	-	-	-	-
Ascomycota	Sordariomycetes	Sordariales				1.19	-	0.22	0.23	0.09	-	-	0.26	-	0.04	-	0.46	-	0.45	0.35	2.71	0.20	0.33	0.32	0.13
Ascomycota	Sordariomycetes	Sordariales				-	0.04	-	-	-	-	-	-	-	-	-	-	0.04	-	-	-	-	-	-	-
Ascomycota	Sordariomycetes	Sordariales				-	-	-	-	-	-	-	-	-	-	-	-	-	-	-	-	-	-	-	0.25
Ascomycota	Sordariomycetes	Sordariales				-	-	-	-	-	-	0.22	-	-	-	-	-	-	-	-	-	-	-	-	-
Ascomycota	Sordariomycetes	Sordariales				-	-	-	-	-	-	-	-	-	-	-	-	-	-	-	-	-	-	0.11	-
Ascomycota	Sordariomycetes	Sordariales				-	-	-	-	-	-	-	-	-	-	-	-	-	-	-	-	-	0.10	-	-
Basidiomycota	Agaricomycetes	Agaricales	Bolbitiaceae	*Panaeolina*	*Panaeolina foenisecii*	-	-	-	0.03	-	-	-	-	-	-	-	-	-	-	-	-	-	-	-	-
Basidiomycota	Agaricomycetes	Agaricales	Clavariaceae	*Clavaria*	*Clavaria californica*	-	-	-	-	-	-	-	-	-	-	-	-	-	-	0.23	-	-	-	-	-
Basidiomycota	Agaricomycetes	Agaricales	Clavariaceae	*Clavaria*	*Clavaria flavipes*	-	-	-	-	-	-	-	-	-	-	12.46	-	-	-	-	-	-	3.18	-	-
Basidiomycota	Agaricomycetes	Agaricales	Clavariaceae	*Clavaria*	*Clavaria flavostellifera*	-	-	-	-	-	-	-	-	-	-	0.19	-	-	0.11	-	-	-	0.10	-	-
Basidiomycota	Agaricomycetes	Agaricales	Clavariaceae	*Clavaria*		-	-	-	-	5.48	-	-	-	0.19	-	-	-	-	-	-	-	-	-	0.52	-
Basidiomycota	Agaricomycetes	Agaricales	Clavariaceae	*Clavaria*		-	-	-	-	0.17	-	-	-	0.22	-	-	-	-	-	-	-	-	-	-	-
Basidiomycota	Agaricomycetes	Agaricales	Clavariaceae	*Clavaria*		0.63	-	0.08	0.04	0.43	-	-	0.25	-	0.12	0.11	0.38	0.24	0.04	0.29	0.13	-	0.50	1.05	0.06
Basidiomycota	Agaricomycetes	Agaricales	Clavariaceae	*Clavaria*		-	-	-	-	-	-	0.04	-	-	-	-	-	-	-	-	-	-	-	-	-
Basidiomycota	Agaricomycetes	Agaricales	Clavariaceae	*Clavicorona*	*Clavicorona taxophila*	0.22	-	-	-	-	-	-	-	-	-	-	-	-	0.06	-	-	-	-	-	-
Basidiomycota	Agaricomycetes	Agaricales	Clavariaceae	*Clavulinopsis*		-	-	-	-	-	-	-	-	-	-	-	-	-	-	-	-	-	-	3.22	-
Basidiomycota	Agaricomycetes	Agaricales	Clavariaceae	*Hodophilus*	*Hodophilus foetens*	-	-	-	-	-	-	-	-	-	-	-	-	-	-	-	-	-	-	0.14	-
Basidiomycota	Agaricomycetes	Agaricales	Clavariaceae			-	-	-	-	-	-	-	-	-	-	2.13	-	-	-	-	-	-	-	-	-
Basidiomycota	Agaricomycetes	Agaricales	Clavariaceae			-	0.13	-	-	-	-	-	-	-	0.12	-	0.97	-	-	-	-	-	0.23	0.95	0.29
Basidiomycota	Agaricomycetes	Agaricales	Clavariaceae			-	-	-	-	-	-	-	-	-	-	0.12	-	-	-	-	-	-	-	-	-
Basidiomycota	Agaricomycetes	Agaricales	Clavariaceae			-	-	-	-	-	-	-	-	-	-	0.06	-	-	-	-	-	-	-	-	-
Basidiomycota	Agaricomycetes	Agaricales	Cortinariaceae	*Cortinarius*	*Cortinarius cinereobrunneolus*	-	-	-	-	-	-	-	-	0.14	-	-	-	-	-	-	-	-	-	-	-
Basidiomycota	Agaricomycetes	Agaricales	Cortinariaceae	*Cortinarius*		-	-	-	-	-	-	-	-	0.16	-	-	-	-	-	-	-	-	-	-	-
Basidiomycota	Agaricomycetes	Agaricales	Hygrophoraceae	*Arrhenia*	*Arrhenia lobata*	0.48	-	0.16	-	-	-	-	0.08	-	-	-	-	-	-	-	-	-	-	-	-
Basidiomycota	Agaricomycetes	Agaricales	Hygrophoraceae	*Cuphophyllus*	*Cuphophyllus fornicatus*	-	-	-	-	-	-	-	-	3.22	-	-	-	-	-	-	-	-	-	-	-
Basidiomycota	Agaricomycetes	Agaricales	Hygrophoraceae	*Cuphophyllus*		-	-	-	-	3.12	-	-	-	-	-	-	-	-	0.30	-	-	-	-	-	-
Basidiomycota	Agaricomycetes	Agaricales	Hygrophoraceae	*Cuphophyllus*		-	-	-	-	-	-	-	-	-	-	-	9.86	-	-	-	-	-	-	2.92	-
Basidiomycota	Agaricomycetes	Agaricales	Hygrophoraceae	*Hygrocybe*	*Hygrocybe conica*	0.69	0.16	26.85	-	-	-	0.03	12.37	-	0.31	25.33	1.05	-	0.17	1.01	0.28	-	0.76	0.12	7.12
Basidiomycota	Agaricomycetes	Agaricales	Hygrophoraceae	*Hygrocybe*		-	-	-	-	-	-	-	-	0.72	-	-	-	-	-	-	-	-	-	-	-
Basidiomycota	Agaricomycetes	Agaricales	Hygrophoraceae			-	78.46	-	-	-	-	-	-	-	0.85	-	13.88	28.55	-	-	-	-	16.20	5.06	-
Basidiomycota	Agaricomycetes	Agaricales	Marasmiaceae			-	-	-	-	-	-	-	-	0.07	-	-	-	-	-	-	-	-	-	-	-
Basidiomycota	Agaricomycetes	Agaricales	Mycenaceae	*Hemimycena*		-	-	-	-	-	-	-	0.05	-	-	-	-	-	-	-	0.95	-	-	-	-
Basidiomycota	Agaricomycetes	Agaricales	Mycenaceae	*Mycena*	*Mycena epipterygia*	-	-	-	-	-	-	-	-	-	85.62	-	-	-	-	-	-	-	-	-	-
Basidiomycota	Agaricomycetes	Agaricales	Pluteaceae	*Pluteus*	*Pluteus cervinus*	-	-	-	-	-	-	-	-	-	-	-	-	-	0.54	-	-	-	-	-	-
Basidiomycota	Agaricomycetes	Agaricales	Pluteaceae	*Pluteus*	*Pluteus ephebeus*	-	-	-	-	-	-	-	-	-	-	-	-	0.07	-	-	-	-	-	-	-
Basidiomycota	Agaricomycetes	Agaricales	Pluteaceae	*Pluteus*	*Pluteus liliputianus*	-	-	-	0.10	-	-	-	-	-	-	-	-	-	-	-	-	-	-	-	-
Basidiomycota	Agaricomycetes	Agaricales	Pluteaceae	*Pluteus*		-	-	-	-	-	-	-	-	-	0.06	-	-	-	-	0.06	-	-	0.16	-	0.36
Basidiomycota	Agaricomycetes	Agaricales	Pluteaceae	*Pluteus*		-	-	-	-	-	-	-	-	-	-	-	-	-	-	-	-	-	0.19	-	0.10
Basidiomycota	Agaricomycetes	Agaricales	Pluteaceae	*Pluteus*		-	-	-	0.13	-	-	-	-	-	-	-	-	-	-	-	-	-	-	-	-
Basidiomycota	Agaricomycetes	Agaricales	Psathyrellaceae	*Coprinellus*		-	-	-	-	-	-	0.12	-	-	-	-	-	-	-	-	-	-	-	-	-
Basidiomycota	Agaricomycetes	Agaricales	Psathyrellaceae	*Coprinopsis*	*Coprinopsis argentea*	-	-	-	-	-	-	-	-	-	-	-	0.06	-	-	-	0.06	-	-	-	-
Basidiomycota	Agaricomycetes	Agaricales	Psathyrellaceae	*Psathyrella*	*Psathyrella typhae*	-	-	-	0.32	-	-	-	0.24	-	-	-	0.17	-	-	-	-	-	-	-	0.05
Basidiomycota	Agaricomycetes	Agaricales	Psathyrellaceae	*Psathyrella*		-	-	-	2.74	-	-	-	0.48	-	-	-	-	-	-	-	-	-	-	-	-
Basidiomycota	Agaricomycetes	Agaricales	Psathyrellaceae			-	0.14	1.20	-	-	-	-	-	-	-	0.34	0.12	0.12	-	0.07	0.08	0.15	-	0.26	-
Basidiomycota	Agaricomycetes	Agaricales	Psathyrellaceae			-	-	-	-	-	-	-	-	-	-	-	-	-	-	-	-	0.27	-	-	-
Basidiomycota	Agaricomycetes	Agaricales	Psathyrellaceae			-	-	-	-	-	-	-	0.54	-	-	-	-	-	-	-	-	-	-	-	-
Basidiomycota	Agaricomycetes	Agaricales	Psathyrellaceae			-	-	-	-	0.11	-	-	-	-	-	-	-	-	-	-	-	-	-	-	-
Basidiomycota	Agaricomycetes	Agaricales	Tricholomataceae	*Dermoloma*		-	-	-	-	-	-	-	-	-	-	0.38	-	-	0.27	-	-	-	0.34	-	-
Basidiomycota	Agaricomycetes	Agaricales	Tricholomataceae	*Melanoleuca*	*Melanoleuca polioleuca*	0.05	-	-	-	-	-	-	-	-	-	-	-	-	-	-	-	-	-	-	-
Basidiomycota	Agaricomycetes	Agaricales	Tricholomataceae	*Tricholoma*	*Tricholoma scalpturatum*	-	-	-	-	-	-	-	-	0.64	-	-	-	-	-	-	-	-	-	-	-
Basidiomycota	Agaricomycetes	Agaricales	Tricholomataceae			-	-	-	-	-	-	0.06	-	-	-	-	-	-	-	-	-	-	-	-	-
Basidiomycota	Agaricomycetes	Agaricales	Tricholomataceae			-	-	-	-	-	-	-	-	0.13	-	-	-	-	-	-	-	-	-	-	-
Basidiomycota	Agaricomycetes	Agaricales				2.02	-	19.76	8.09	-	-	23.49	1.82	-	-	-	-	-	37.15	0.84	0.80	0.47	-	-	-
Basidiomycota	Agaricomycetes	Agaricales				-	-	-	-	-	-	-	-	-	0.28	2.69	-	-	-	-	-	-	0.09	-	-
Basidiomycota	Agaricomycetes	Agaricales				1.77	0.20	-	-	-	-	-	0.30	-	-	0.20	-	-	0.04	0.86	-	0.09	0.22	0.50	0.28
Basidiomycota	Agaricomycetes	Agaricales				-	-	-	0.91	-	-	-	-	-	-	-	-	-	-	-	-	-	-	-	-
Basidiomycota	Agaricomycetes	Agaricales				-	-	-	0.05	-	-	-	-	-	-	-	-	-	-	1.12	-	-	-	-	-
Basidiomycota	Agaricomycetes	Agaricales				-	-	-	-	0.13	-	-	-	-	-	-	-	-	0.13	-	-	-	-	-	-
Basidiomycota	Agaricomycetes	Agaricales				0.32	-	-	-	-	-	-	-	-	-	0.08	-	-	-	-	-	-	0.32	-	-
Basidiomycota	Agaricomycetes	Cantharellales	Ceratobasidiaceae	*Ceratobasidium*		-	-	-	-	-	-	-	0.59	-	-	0.13	0.07	-	-	-	-	-	-	0.24	-
Basidiomycota	Agaricomycetes	Cantharellales	Ceratobasidiaceae	*Ceratobasidium*		0.07	-	-	-	-	-	-	-	-	-	-	-	-	-	-	-	-	-	-	-
Basidiomycota	Agaricomycetes	Cantharellales	Ceratobasidiaceae	*Ceratobasidium*		-	-	-	-	-	-	-	-	-	-	0.11	-	-	-	-	-	-	-	-	-
Basidiomycota	Agaricomycetes	Cantharellales	Ceratobasidiaceae	*Ceratobasidium*		0.09	-	-	-	-	-	-	-	-	-	-	-	-	-	-	-	-	-	-	-
Basidiomycota	Agaricomycetes	Cantharellales	Ceratobasidiaceae	*Ceratobasidium*		-	-	-	-	-	-	-	-	-	-	-	-	0.04	-	-	-	-	-	-	-
Basidiomycota	Agaricomycetes	Cantharellales	Ceratobasidiaceae			0.17	-	-	-	-	-	-	-	-	-	-	-	-	-	-	-	-	-	-	-

Basidiomycota	Agaricomycetes	Cantharellales	Ceratobasidiaceae			-	-	-	-	-	-	-	0.09	-	-	-	-	-	-	-	-	-	-	-	-
Basidiomycota	Agaricomycetes	Cantharellales	Ceratobasidiaceae			-	-	-	-	-	-	0.04	-	-	-	-	-	-	-	-	-	-	-	-	-
Basidiomycota	Agaricomycetes	Cantharellales	Ceratobasidiaceae			-	-	-	-	-	-	-	-	-	-	0.07	-	-	-	-	-	-	-	-	-
Basidiomycota	Agaricomycetes	Cantharellales	Tulasnellaceae	*Tulasnella*		-	-	-	-	-	95.46	-	-	-	-	-	-	-	-	-	-	-	-	-	-
Basidiomycota	Agaricomycetes	Sebacinales	Sebacinaceae	*Helvellosebacina*	*Helvellosebacina helvelloides*	-	-	-	-	0.55	-	-	-	-	-	-	-	-	-	-	-	-	-	-	-
Basidiomycota	Agaricomycetes	Sebacinales	Sebacinaceae	*Sebacina*		-	-	-	-	-	-	-	-	8.21	-	-	-	-	-	-	-	-	-	-	-
Basidiomycota	Agaricomycetes	Sebacinales	Sebacinaceae	*Sebacina*		0.13	0.04	0.64	-	-	-	-	0.20	-	-	-	0.50	0.14	-	-	-	-	0.10	0.64	-
Basidiomycota	Agaricomycetes	Sebacinales	Sebacinaceae	*Sebacina*		-	-	-	-	-	-	-	-	-	-	-	-	-	-	-	-	-	0.06	-	0.11
Basidiomycota	Agaricomycetes	Sebacinales	Sebacinaceae	*Sebacina*		-	-	-	-	-	-	-	-	-	-	-	-	-	-	-	-	-	0.10	-	-
Basidiomycota	Agaricomycetes	Sebacinales	Sebacinaceae	*Sebacina*		-	-	0.08	-	-	-	-	-	-	-	-	-	-	-	-	-	-	-	-	-
Basidiomycota	Agaricomycetes	Sebacinales	Sebacinaceae	*Sebacina*		-	-	-	-	-	-	-	-	-	-	-	-	-	-	-	-	-	0.11	-	-
Basidiomycota	Agaricomycetes	Sebacinales	Sebacinaceae			1.38	-	-	-	-	-	-	0.12	-	-	-	-	-	-	-	-	-	-	-	-
Basidiomycota	Agaricomycetes	Sebacinales	Sebacinaceae			-	-	-	-	-	-	-	-	-	-	-	-	0.82	-	-	-	-	-	0.06	-
Basidiomycota	Agaricomycetes	Sebacinales	Sebacinaceae			0.07	0.14	0.38	-	-	-	-	-	-	-	0.73	0.12	0.11	-	-	0.50	-	0.21	0.87	-
Basidiomycota	Agaricomycetes	Sebacinales	Sebacinaceae			0.06	-	0.12	-	-	-	-	-	-	-	-	-	-	-	-	-	-	-	-	-
Basidiomycota	Agaricomycetes	Sebacinales	Sebacinaceae			0.07	-	-	0.08	-	-	-	0.10	-	-	-	-	-	0.18	0.43	0.31	-	-	0.05	-
Basidiomycota	Agaricomycetes	Sebacinales	Sebacinaceae			-	-	-	-	-	-	-	-	-	-	0.05	-	-	-	-	-	-	-	-	-
Basidiomycota	Agaricomycetes	Sebacinales	Sebacinaceae			-	-	-	-	-	-	-	-	-	-	0.05	0.11	-	-	-	-	-	-	-	-
Basidiomycota	Agaricomycetes	Sebacinales	Sebacinaceae			-	-	-	-	-	-	-	-	-	-	-	-	-	0.05	-	-	-	-	-	-
Basidiomycota	Agaricomycetes	Sebacinales	Sebacinaceae			-	-	-	-	-	-	-	0.10	-	-	-	-	-	-	-	-	-	-	-	-
Basidiomycota	Agaricomycetes	Sebacinales				-	-	0.86	0.34	-	-	-	0.25	-	-	-	-	-	0.04	-	-	-	0.50	-	0.08
Basidiomycota	Agaricomycetes	Sebacinales				-	0.05	-	-	-	-	-	-	-	-	-	-	0.34	-	-	-	-	-	-	-
Basidiomycota	Agaricomycetes	Sebacinales				-	-	-	-	-	-	-	-	-	0.04	-	0.15	-	0.04	0.09	-	-	-	-	-
Basidiomycota	Agaricomycetes	Sebacinales				-	-	-	-	-	-	-	-	-	-	-	-	-	-	-	-	0.15	-	-	-
Basidiomycota	Agaricomycetes	Sebacinales				-	-	-	-	-	-	-	-	-	-	-	0.09	-	-	-	-	-	-	-	-
Basidiomycota	Agaricomycetes	Thelephorales	Thelephoraceae	*Tomentella*	*Tomentella fuscocinerea*	-	-	-	-	-	-	-	-	1.35	-	-	-	-	-	-	-	-	-	-	-
Basidiomycota	Agaricomycetes	Thelephorales	Thelephoraceae	*Tomentella*	*Tomentella testaceogilva*	-	-	0.42	0.35	-	-	0.56	-	-	-	-	-	-	6.25	-	0.55	-	-	-	-
Basidiomycota	Agaricomycetes	Thelephorales	Thelephoraceae	*Tomentella*		-	-	-	-	14.57	-	-	-	5.62	-	-	-	-	-	-	-	-	-	-	-
Basidiomycota	Agaricomycetes	Thelephorales	Thelephoraceae	*Tomentella*		-	-	-	-	12.66	-	-	-	0.39	-	-	-	-	-	-	-	-	-	-	-
Basidiomycota	Agaricomycetes	Thelephorales	Thelephoraceae	*Tomentella*		-	-	-	-	0.31	-	-	-	0.33	-	-	-	-	-	-	-	-	-	-	-
Basidiomycota	Agaricomycetes	Thelephorales	Thelephoraceae	*Tomentella*		-	-	-	-	-	-	-	-	9.41	-	-	-	-	-	-	-	-	-	-	-
Basidiomycota	Agaricomycetes	Thelephorales	Thelephoraceae	*Tomentella*		-	-	-	-	0.08	-	-	-	1.50	-	-	-	-	-	-	-	-	-	-	-
Basidiomycota	Agaricomycetes	Thelephorales	Thelephoraceae	*Tomentella*		2.77	-	0.18	-	-	-	-	1.82	-	0.22	-	-	0.23	1.79	0.31	0.43	0.04	0.06	-	-
Basidiomycota	Agaricomycetes	Thelephorales	Thelephoraceae	*Tomentella*		-	-	-	-	-	-	-	-	3.76	-	-	-	-	-	-	-	-	-	-	-
Basidiomycota	Agaricomycetes	Thelephorales	Thelephoraceae	*Tomentella*		0.19	-	2.61	0.08	-	-	-	-	-	-	-	0.06	-	0.16	-	2.40	-	-	-	-
Basidiomycota	Agaricomycetes	Thelephorales	Thelephoraceae	*Tomentella*		-	-	-	-	-	-	-	-	-	-	-	-	-	0.75	-	-	-	-	-	-
Basidiomycota	Agaricomycetes	Thelephorales	Thelephoraceae	*Tomentella*		-	-	-	-	0.10	-	-	-	0.82	-	-	-	-	-	-	-	-	-	-	-
Basidiomycota	Agaricomycetes	Thelephorales	Thelephoraceae	*Tomentella*		-	-	-	-	-	-	-	-	0.24	-	-	-	-	-	-	-	-	-	-	-
Basidiomycota	Agaricomycetes	Thelephorales	Thelephoraceae	*Tomentella*		-	-	-	-	-	-	-	-	-	-	-	-	-	-	-	0.14	-	-	-	-
Basidiomycota	Agaricomycetes	Thelephorales	Thelephoraceae			-	-	-	-	28.49	-	-	-	10.92	-	-	-	-	-	-	-	-	-	-	-
Basidiomycota	Agaricomycetes	Thelephorales	Thelephoraceae			-	-	-	0.31	-	-	-	-	-	-	-	-	-	-	-	-	-	-	-	-
Basidiomycota	Agaricomycetes	Thelephorales	Thelephoraceae			-	-	-	-	0.08	-	-	-	-	-	-	-	-	-	-	-	-	-	-	-
Basidiomycota	Tremellomycetes	Tremellales	Bulleribasidiaceae			1.20	0.13	1.00	3.15	-	-	1.92	1.53	-	0.33	3.65	1.17	0.54	0.26	0.86	0.62	0.88	1.18	6.08	3.79
Basidiomycota	Tremellomycetes	Tremellales	Bulleribasidiaceae			-	-	-	0.09	-	-	-	-	-	-	0.54	-	-	-	0.04	-	-	0.06	0.08	-
Basidiomycota	Tremellomycetes	Tremellales	Bulleribasidiaceae			-	-	-	-	-	-	0.05	-	-	-	-	-	-	-	-	-	-	-	-	-
Basidiomycota	Tremellomycetes	Tremellales	Tremellaceae	*Dioszegia*	*Dioszegia fristingensis*	-	-	-	0.10	-	-	0.10	-	-	-	0.08	0.06	-	-	0.08	0.06	-	0.09	0.60	0.11
Basidiomycota	Tremellomycetes	Tremellales	Tremellaceae	*Dioszegia*	*Dioszegia fristingensis*	0.04	-	-	0.05	-	-	0.06	-	-	-	-	-	-	-	-	-	-	-	0.13	0.11
Basidiomycota	Tremellomycetes	Tremellales	Tremellaceae	*Dioszegia*	*Dioszegia hungarica*	-	-	-	-	-	-	-	-	-	-	-	-	-	-	-	-	-	-	0.13	-
Basidiomycota	Tremellomycetes	Tremellales	Tremellaceae	*Dioszegia*	*Dioszegia rishiriensis*	-	-	-	0.06	-	-	-	-	-	-	-	-	-	-	-	-	-	0.06	0.15	-
Basidiomycota	Tremellomycetes	Tremellales	Tremellaceae	*Papiliotrema*	*Papiliotrema frias*	0.06	-	-	0.11	-	-	0.07	0.10	-	-	0.14	-	-	-	0.08	-	-	0.06	0.51	0.12
Basidiomycota	Tremellomycetes	Tremellales				0.21	0.48	0.21	0.93	-	-	0.05	0.24	-	-	0.64	0.28	0.09	0.03	0.09	0.14	0.09	0.12	0.38	0.33
Basidiomycota	Tremellomycetes	Tremellales				-	-	-	0.07	-	-	0.05	-	-	-	0.07	-	-	-	0.04	-	-	0.10	0.32	0.18
Basidiomycota	Tremellomycetes	Tremellales				-	-	-	0.06	-	-	-	-	-	-	0.05	-	-	-	-	-	-	-	-	-
Basidiomycota	Tremellomycetes	Tremellales				-	-	-	-	-	-	-	-	-	-	-	-	-	-	-	-	-	-	0.06	-
Basidiomycota	Tremellomycetes	Tremellales				-	-	-	-	-	-	0.03	-	-	-	-	-	-	-	-	-	-	-	-	-
Glomeromycota	Glomeromycetes	Glomerales	Claroideoglomeraceae	*Claroideoglomus*	*Claroideoglomus claroideum*	0.06	-	-	-	-	-	-	-	-	-	-	-	-	-	-	-	-	-	-	-
Glomeromycota	Glomeromycetes	Glomerales	Glomeraceae	*Dominikia*		-	-	-	-	-	-	-	0.05	-	-	-	-	-	-	-	0.04	-	-	-	-
Glomeromycota	Glomeromycetes	Glomerales	Glomeraceae	*Funneliformis*	*Funneliformis mosseae*	-	-	-	-	-	-	0.28	-	-	-	-	-	-	-	-	-	-	-	-	-
Glomeromycota	Glomeromycetes	Glomerales	Glomeraceae	*Glomus*		-	-	-	-	-	-	-	-	0.09	-	-	-	-	-	-	-	-	-	-	-
Glomeromycota	Glomeromycetes	Glomerales	Glomeraceae	*Glomus*		-	-	-	-	-	-	-	0.11	-	-	0.09	-	-	-	-	-	-	0.15	0.12	0.05
Glomeromycota	Glomeromycetes	Glomerales	Glomeraceae	*Glomus*		-	-	-	-	-	-	-	0.06	-	-	-	-	-	-	-	-	-	0.06	-	-
Glomeromycota	Glomeromycetes	Glomerales	Glomeraceae			-	-	-	-	-	-	-	-	-	-	-	-	-	-	-	-	0.13	-	-	-
Glomeromycota	Glomeromycetes	Glomerales	Glomeraceae			-	-	-	-	-	-	0.07	-	-	0.04	-	-	-	-	-	-	-	-	-	-
Glomeromycota	Glomeromycetes	Glomerales	Glomeraceae			-	-	-	-	-	-	0.07	-	-	-	-	-	-	-	-	-	-	-	-	-
Glomeromycota	Glomeromycetes	Glomerales	Glomeraceae			-	-	0.07	-	-	-	-	-	-	-	-	-	-	-	0.10	0.14	-	-	-	-
Glomeromycota	Glomeromycetes	Glomerales	Glomeraceae			0.07	-	-	-	-	-	-	-	-	-	-	-	-	-	-	-	-	-	-	-
Glomeromycota	Glomeromycetes	Glomerales	Glomeraceae			-	-	-	-	-	-	-	-	-	-	0.04	0.12	-	-	-	-	-	-	0.05	-
Zygomycota	Mortierellomycetes	Mortierellales	Mortierellaceae	*Mortierella*	*Mortierella alpina*	-	-	-	-	-	-	-	-	0.04	-	-	-	-	-	-	-	-	-	-	-
Zygomycota	Mortierellomycetes	Mortierellales	Mortierellaceae			0.05	-	-	0.03	-	-	0.08	-	-	-	-	-	-	-	-	-	-	-	-	-
Zygomycota	Mortierellomycetes	Mortierellales	Mortierellaceae			0.22	-	-	0.04	-	-	0.07	-	-	-	0.07	-	-	-	0.04	0.07	-	0.10	-	0.08
Zygomycota	Mortierellomycetes	Mortierellales	Mortierellaceae			-	-	-	-	-	-	0.13	-	-	-	-	-	-	0.04	-	-	-	-	-	-
Zygomycota	Mortierellomycetes	Mortierellales	Mortierellaceae			0.17	-	-	0.09	-	-	0.16	0.05	-	-	-	-	-	-	-	-	0.05	-	-	-
Zygomycota	Mortierellomycetes	Mortierellales	Mortierellaceae			-	-	0.19	-	-	-	-	-	-	-	-	-	-	-	-	-	-	-	-	-
Zygomycota	Mortierellomycetes	Mortierellales	Mortierellaceae			-	-	-	-	-	-	-	-	0.03	-	-	-	-	-	-	-	-	-	-	-

Table S3. Soil analysis results showing p values from Pearson Correlation analysis of all plots studied, orchid hosting plots, and non-orchid hosting plots

	Shan Index	Sim Index	NO3	PO4	Cantharellales	Sebacinales	Thelephorales	Capnodiales	Pleosporales	Chaetothyriales	Helotiales	Pezizales	Hypocreales	Sordariales	Agaricales	Tremellales	Glomerales	Mortierellales
Shan Index		3.68259E-11	0.445385858	0.445385857	0.782041433	0.847264491	0.805795917	0.844784982	0.845544352	0.328694149	0.131194218	0.627956935	0.494572395	0.740901278	0.351414259	0.979537345	0.985338506	0.112637373
Sim Index			0.660840709	0.660840708	0.982014758	0.926721343	0.554357113	0.63854183	0.393420772	0.172280217	0.078904586	0.640981413	0.792119539	0.876339742	0.794575394	0.999052883	0.995254984	0.137262087
NO3					0.003080209	0.650097679	0.69729924	0.991529263	0.416700106	0.567844084	0.117566135	0.029287941	0.077108513	0.024330318	0.485949212	0.887319593	0.937700915	0.633317258
PO4					0.003080209	0.650097679	0.69729924	0.991529263	0.416700106	0.567844084	0.117566135	0.029287941	0.077108513	0.024330318	0.485949212	0.887319593	0.937700915	0.633317258
Cantharellales						0.55994606	0.679763015	0.999258307	0.602847674	0.408471277	0.027881857	0.11209498	0.096009144	0.497462331	0.991970235	0.853062977	0.799056644	0.522167455
Sebacinales							0.390496782	0.568962672	0.316012568	0.851040374	0.562190882	0.812588714	0.222774394	0.247220541	0.432789095	0.640676853	0.642184586	0.630090243
Thelephorales								0.685564825	0.085131902	0.39791334	0.177950032	0.517912309	0.138013995	0.242731262	0.706054968	0.21624032	0.207417482	0.58700184
Capnodiales									0.01555476	0.044557779	0.470245236	0.462846811	0.197001197	0.754187319	0.957378815	0.039146236	0.037908381	0.292975061
Pleosporales										0.067334858	0.336823591	0.246217012	0.006425808	0.000438513	0.140058839	0.168799668	0.161172297	0.999302154
Chaetothyriales											0.596810695	0.93898536	0.240584752	0.588721677	0.155905139	0.056528595	0.058552255	0.376470769
Helotiales												0.654587786	0.274917808	0.751694788	0.969480713	0.572649581	0.547555084	0.339126952
Pezizales													0.001002914	0.006727267	0.204479629	0.703102327	0.732350033	0.519113559
Hypocreales														9.78465E-05	0.112214529	0.087799541	0.078061223	0.761626
Sordariales															0.049955749	0.895584654	0.861920047	0.737072723
Agaricales																0.658838497	0.645885532	0.283673551
Tremellales																	6.05882E-31	0.942799504
Glomerales																		0.938116749

ORCHID PLOTS

	Shan Index	Sim Index	NO3	PO4	Cantharellales	Sebacinales	Thelephorales	Capnodiales	Pleosporales	Chaetothyriales	Helotiales	Pezizales	Hypocreales	Sordariales	Agaricales	Tremellales	Glomerales	Mortierellales
Shan Index		0.005559388	0.329093895	0.329093895	0.18178174	0.623930587	0.414136821	0.677163465	0.981177122	0.178831227	0.270561401	0.825878272	0.625720046	0.983333661	0.561301195	0.890291843	0.894588306	0.448817728
Sim Index			0.512594789	0.512594789	0.304165605	0.577448308	0.344965911	0.647302674	0.886315193	0.165188234	0.213804136	0.809342323	0.579068643	0.846394836	0.379125576	0.853770286	0.850947645	0.614925238
NO3				0	0.369656718	0.876625364	0.644012717	0.342359495	0.608780182	0.34506942	0.947514231	0.926895087	0.765671166	0.976909933	0.969294179	0.068820336	0.068656977	0.721784274
PO4					0.369656718	0.876625364	0.644012717	0.342359495	0.608780182	0.34506942	0.947514231	0.926895087	0.765671166	0.976909933	0.969294179	0.068820336	0.068656977	0.721784274
Cantharellales						0.588770522	0.526373133	0.978599885	0.512438981	0.354899471	0.964339351	0.396734479	0.456671347	0.976322971	0.966784649	0.725607969	0.73305542	0.142760307
Sebacinales							0.509540409	0.897635146	0.854606128	0.758332096	0.051495603	0.065821941	0.21687029	0.35695122	0.897325817	0.906917832	0.909102016	0.995771743
Thelephorales								0.138631634	0.340830949	0.017111241	0.791126701	0.103360257	0.014663622	0.049977398	0.054322481	0.801488035	0.808653115	0.627165933
Capnodiales									0.025325514	0.128979201	0.778356	0.517942068	0.264389912	0.153048009	0.179935966	0.624660434	0.6136859	0.269781207
Pleosporales										0.374921365	0.872867039	0.835130791	0.531082719	0.223856589	0.211389911	0.768768868	0.755800693	0.07679631
Chaetothyriales											0.948894971	0.241330525	0.083097413	0.202496638	0.130006701	0.921134313	0.916147805	0.859827684
Helotiales												0.29502044	0.476260362	0.451681975	0.893006488	0.766676912	0.76133919	0.664931326
Pezizales													0.013402133	0.116420145	0.365554715	0.74586349	0.749717799	0.911466555
Hypocreales														0.044872761	0.160133819	0.784250592	0.79043332	0.724447333
Sordariales															0.065471733	0.651391122	0.662094808	0.245170098
Agaricales																0.461468739	0.469838872	0.316509093
Tremellales																	6.04312E-07	0.785045453
Glomerales																		0.798500293

NON-ORCHID PLOTS ONLY

	Shan Index	Sim Index	NO3	PO4	Cantharellales	Sebacinales	Thelephorales	Capnodiales	Pleosporales	Chaetothyriales	Helotiales	Pezizales	Hypocreales	Sordariales	Agaricales	Tremellales	Glomerales	Mortierellales
Shan Index		2.54595E-07	0.516939677	0.516939677	0.540123955	0.737657356	0.898350575	0.407320013	0.585132029	0.15655166	0.950177496	0.705359073	0.490825318	0.85175182	0.218996165	0.986102773	0.989253486	0.001276148
Sim Index			0.6645396	0.6645396	0.71171436	0.811002917	0.553640076	0.537746814	0.84210056	0.260207851	0.939346939	0.713189852	0.862163959	0.711858307	0.576601142	0.799820639	0.795081081	0.000386866
NO3				0	0.007868855	0.646641448	0.644505785	0.705313875	0.411789774	0.192089125	0.015929997	0.051678517	0.121293418	0.029926422	0.534724949	0.531701205	0.571259654	0.59680196
PO4					0.007868855	0.646641447	0.644505785	0.705313875	0.411789774	0.192089125	0.015929997	0.051678517	0.121293418	0.029926422	0.534724949	0.531701205	0.571259654	0.596801961
Cantharellales						0.582623202	0.659857903	0.893828903	0.710965846	0.403330745	0.000261837	0.186684179	0.154774369	0.572658908	0.963317893	0.871835456	0.822123051	0.470787936
Sebacinales							0.430054448	0.610588142	0.331296256	0.615778298	0.328538033	0.826795002	0.264387703	0.304432628	0.500837319	0.641404882	0.641429398	0.591761766
Thelephorales								0.576373343	0.066304685	0.190936314	0.150238006	0.472908014	0.085907716	0.182103759	0.594100654	0.240993881	0.229436353	0.488744721
Capnodiales									0.286235273	0.624080791	0.192636178	0.380533024	0.241426852	0.788214379	0.690248069	0.028658794	0.028000603	0.265660762
Pleosporales										0.36252528	0.233515471	0.154243518	0.000324702	7.06855E-05	0.17927189	0.13903951	0.132083821	0.844382358
Chaetothyriales											0.923580419	0.948460462	0.313048597	0.984796053	0.198146833	0.002418826	0.002632846	0.481456243
Helotiales												0.23205689	0.006723359	0.200085025	0.89208448	0.230168441	0.201718993	0.483474644
Pezizales													0.006869439	0.024247645	0.318602222	0.74770632	0.773802144	0.461649385
Hypocreales														0.001491761	0.251564294	0.065451221	0.05774297	0.573847418
Sordariales															0.148721017	0.769550773	0.741528245	0.925379843
Agaricales																0.521045129	0.51186959	0.226283963
Tremellales																	2.25404E-22	0.944233901
Glomerales																		0.934049356
